# Adipose tissue-derived stromal cells enhance glycolytic metabolism in injured nerve cells via the FOXK1-HK2 axis for spinal cord injury repair

**DOI:** 10.1186/s12967-026-07958-w

**Published:** 2026-03-17

**Authors:** Fang Li, Hongbo Li, Yanfei Jia, Lanxiang Ou, Yuepeng Fang, Liuzhu Pan, Hua Liu, Bin Ning

**Affiliations:** 1 Central Hospital Affiliated to Shandong First Medical University, 105 Jie Fang Road, Jinan, Shandong 250013 China; 2 Shandong Laibo Biotechnology Co., Ltd, Jinan, Shandong China; 3 Jinan Central Hospital, Shandong University, Jinan, Shandong 250013 China; 4 School of Clinical Medicine, Shandong Second Medical University, Weifang, China

**Keywords:** Spinal cord injury, Adipose tissue-derived stromal cells, Glycolysis, HK2, FOXK1

## Abstract

**Background:**

Spinal cord injury (SCI) causes severe energy metabolism dysfunction, hindering neuronal survival and recovery. Adipose tissue-derived stromal cells (ADSCs) have neuroprotective potential, but their role in regulating neuronal energy metabolism and the underlying mechanisms remain unclear. This study aimed to investigate whether ADSCs are capable of restoring neuronal glycolysis through the FOXK1-HK2 signaling pathway, thereby replenishing the energy supply and facilitating tissue regeneration.

**Methods:**

We employed rat and cell models of SCI to observe the effects of ADSCs on glycolytic metabolism and apoptosis. Transcriptome sequencing identified glycolysis-related differentially expressed genes. Lactate detection and Seahorse assays were used to quantify glycolytic activity. Dual-luciferase reporter assays verified the FOXK1-HK2 regulatory relationship. Cut&Run assay provided direct evidence of FOXK1 binding to the HK2 promoter. Behavioral tests, histopathological staining and immunofluorescence were used to evaluate in vivo functional recovery and tissue repair. FOXK1 knockdown confirmed its role in the ADSC-mediated pathway.

**Results:**

We found that ADSCs exerted multiple protective and regulatory effects on neurons and motor function. Specifically, they strongly inhibited neuronal oxidative stress, protected mitochondria, and promoted neuronal metabolic reprogramming. Additionally, ADSCs increased glycolytic activity and lactate production, which further contributed to promoting neuronal survival and the recovery of hindlimb motor function. Blocking TGF-β1 signaling abrogated ADSC-induced activation of the FOXK1-HK2 axis and subsequent enhancement of glycolysis, confirming TGF-β1 as a critical paracrine mediator. Through interaction with HK2, FOXK1 plays a critical role in modulating glycolysis. Dual-luciferase reporter and Cut&Run assays confirmed that FOXK1 regulates the HK2 promoter, thereby increasing its transcriptional activity. The inhibition of FOXK1 expression resulted in suppressed HK2 expression, reduced glycolytic flux, and weakened the neuroprotective effects of ADSCs on SCI.

**Conclusions:**

ADSCs are considered a potential option for SCI treatment, and their therapeutic effects are closely related to the FOXK1/HK2 axis, which mediates ADSCs’ regulation of neuronal glycolytic metabolism to exert protective and reparative functions.

**Graphical Abstract:**

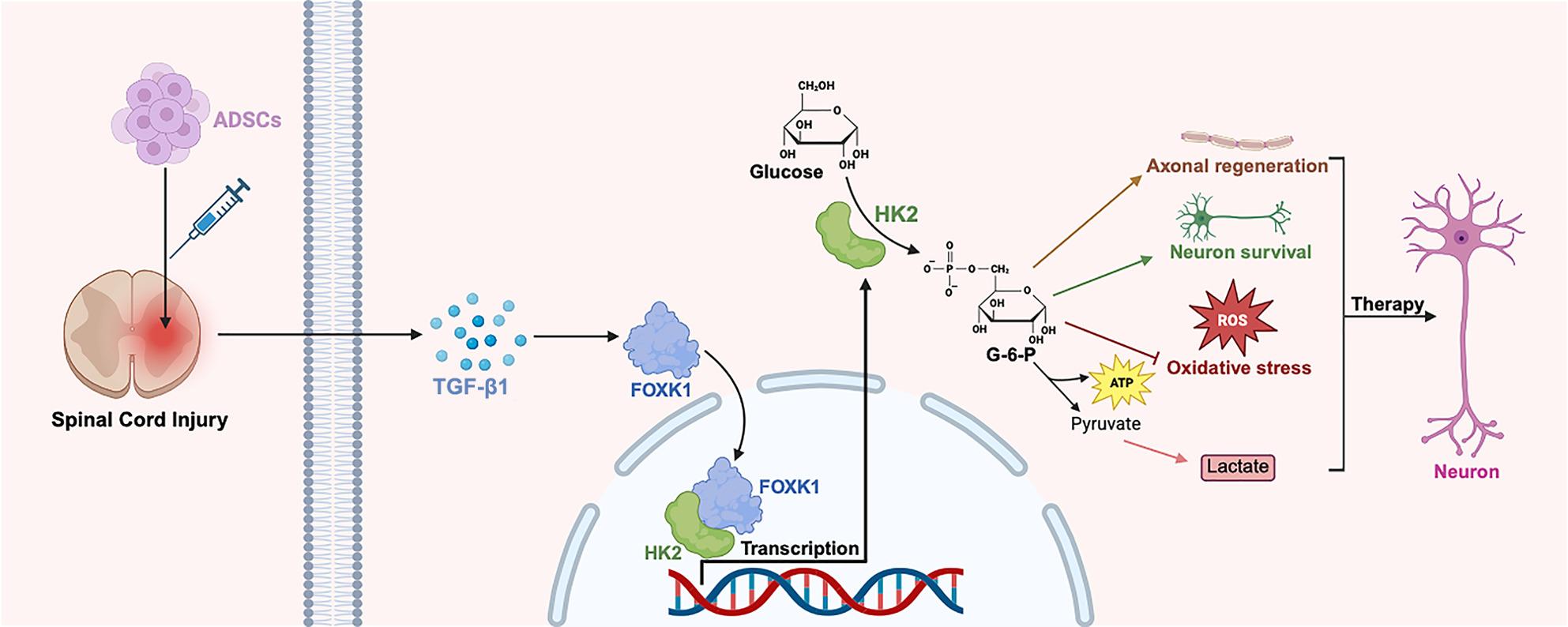

**Supplementary Information:**

The online version contains supplementary material available at 10.1186/s12967-026-07958-w.

## Introduction

Spinal cord injury (SCI) is a severe central nervous system disorder, and although significant advances have been made in SCI treatment, options that can promote neuronal regeneration and functional recovery are still limited [1–3]. Pathologically, SCI involves primary and secondary injuries, both of which are linked to neuronal impairment. Primary injury induces vascular damage and progressive edema, exacerbating ischemia/hypoxia at the injury site, resulting in insufficient neuronal energy production and apoptosis [4]. The reactive oxygen, nitrogen species, and inflammatory cytokines produced during the secondary injury further cause mitochondrial damage and energy deficiency, which aggravate diseases [5].

Energy metabolism disorders in neurons are a core link in the process of neuronal damage and functional recovery obstruction after SCI [6–8]. Therefore, restoring energy metabolism in nerve cells post-SCI has emerged as a crucial strategy for protecting residual neural tissues and promoting recovery of spinal cord function [9, 10]. Mesenchymal stromal cells (MSCs) exhibit antioxidant properties and immunomodulatory capabilities that may enhance their cytoprotective effects on ischemia/reperfusion injury and neurological diseases. These effects include mitigating mitochondrial dysfunction, improving glycolytic efficiency, and enhancing cellular bioenergetics [11–14]. Adipose tissue-derived stromal cells (ADSCs) represent a superior alternative to MSCs derived from other tissues, owing to their lower immunogenicity, abundant availability, and absence of ethical controversies associated with their use [15, 16]. ADSCs have emerged as a regenerative therapy for SCI, mainly because of their neurotrophic effects and immunomodulatory capacities [17–19]. However, whether ADSCs influence energy metabolism after SCI and its regulatory mechanisms remain unclear.

Neurons metabolize glucose through glycolysis in vivo and require glycolysis to maintain ATP levels at the synapses, as well as normal neuronal function [20]. The ATP and lactic acid produced by glycolysis play important roles in neuronal apoptosis and axonal regeneration [21, 22]. This finding provides important insight: since glycolysis can produce key metabolites necessary for neuronal survival and regeneration, promoting glycolytic metabolism in neurons may improve the cellular energy supply, regulate processes related to apoptosis and regeneration, and thus serve as a potentially important regulatory approach to facilitate SCI recovery.

As the first rate-limiting enzyme in glycolysis, hexokinases (HKs) catalyze the phosphorylation of glucose to glucose-6-phosphate. HK2 plays a critical role in governing glycolytic flux and mitochondrial activity, thereby modulating microglial functions in maladaptive inflammation in brain diseases [23, 24]. One study conducted differential gene expression analysis, identified key disulfidptosis-related differentially expressed genes (DRDEGs), and revealed that HK2 plays multiple roles in different pathological stages following SCI [25]. However, how HK2 functions in SCI and its upstream regulatory mechanisms remain unreported. As a member of the FOX family, the transcription factor forkhead box protein K1 (FOXK1) plays fundamental roles in regulating a few cellular processes, including cell proliferation, differentiation, metabolism, and cardiogenesis [26]. FOXK1, a new critical glycolysis regulator, exhibits both fibrogenic properties and metabolism-rewiring capacities during renal fibrosis [27]. Notably, FOXK1 has also been shown to trigger bone formation through the induction of aerobic glycolysis [28], further highlighting its role in regulating glycolytic metabolism. Notably, FOXK1 has been reported to mediated aerobic glycolysis in adipocyte cells by regulating the glycolytic enzymes hexokinase-2 (HK2) and lactate dehydrogenase (LDH) [29]. The role of FOXK1 in SCI, remains largely unexplored.

In this study, by employing cell and mouse models, we revealed that glycolytic metabolism plays a critical role in the promotion of injured neuron repair by ADSCs. Mechanistically, we found that ADSCs promote the repair of injured nerve cells through the FOXK1/HK2 pathway. Notably, by disrupting the FOXK1-HK2 axis, we effectively abrogated the beneficial effects typically conferred by ADSCs in vivo and in vitro, reinforcing the central role of this molecular pathway in mediating the therapeutic effects of stem cell transplantation.

## Materials and methods

### Cell culture

Pheochromocytoma-12 (PC12) cells and human neuroblastoma (SH-SY5Y) cells possess neuronal properties and have been widely employed in vitro studies of SCI [30, 31]. The PC12 neuronal cell line and SH-SY5Y cell line were obtained from Procell Life Science & Technology Co., Ltd. (Wuhan, China). The cells were cultured in a humidified atmosphere with 5% CO_2_ at 37 °C in Dulbecco’s modified Eagle medium/nutrient mixture F-12 (DMEM/F12, Procell, Wuhan, China) supplemented with 10% fetal bovine serum (Corning, NY, USA), 100 U/ml penicillin, and 100 µg/mL streptomycin (100X, Beyotime Biotechnology, Shanghai, China).

The isolation and culture of ADSCs were described in our previous study [32]. ADSCs were isolated from 50-mL aliquots of human adipose tissue harvested via abdominal liposuction procedures. The excised tissue samples were rinsed with phosphate-buffered saline (PBS) to eliminate contaminating red blood cells. Following quantification of the lipid fraction volume, an equivalent volume of 0.2% (w/v) collagenase I solution was supplemented, and the mixture was subjected to enzymatic digestion at 37 °C for 30 min on a shaking incubator maintained at 200 rpm. The resulting cell suspension was filtered through a 70 μm cell strainer to remove undigested tissue debris, after which the filtrated cells were seeded and cultured in Alpha Minimum Essential Medium (α-MEM, Gibco, Grand Island, NY, USA) under a humidified atmosphere of 5% CO_2_ at 37 °C.

### Cell viability assay

Prior to cell processing, an H₂O₂-induced neural cell injury model was established in PC12 and SH-SY5Y cells via treatment with gradient of H₂O₂ concentrations (200–1200 µM for PC12 cells; 50–800 µM for SH-SY5Y cells). Cell viability was assessed via a Cell Counting Kit-8 according to the manufacturer’s protocols [33]. Briefly, PC12 and SH-SY5Y cells were seeded into 96-well plates and incubated with various concentrations of H_2_O_2_ and 2-deoxy-D-glucose (2-DG). Subsequently, 10 µL of Cell Counting Kit-8 solution (GLPBIO, Montclair, CA, USA) was added to each well and incubated for 2 h at 37 °C. The absorbance value of each well at 450 nm was measured via a multifunctional microplate reader (Molecular Devices, SpectraMax i3x, USA).

### Transcriptome sequencing (RNA-seq)

RNA sequencing was performed via a Clariom S expression profile microarray (Genechem Co., Ltd., Shanghai, China) on three groups of PC12 cells: the normal group, H₂O₂-treated group, and ADSCs co-cultured group. Before library preparation, ribosomal RNAs (rRNAs) were depleted from the total RNA. Following quality control and quantification of the library, sequencing was performed on an Illumina HiSeq platform using 150 bp paired-end reads. After sequencing, the raw data were processed and aligned to the reference genome through the STAR aligner.

### Measurement of the mitochondrial membrane potential (MMP)

MMP was measured using an enhanced mitochondrial membrane potential assay kit with JC-1 (Beyotime Biotechnology, Shanghai, China). Briefly, the PC12 and SH-SY5Y cells were incubated with JC-1 staining solution at 37 °C for 30 min, washed twice with JC-1 staining buffer, and then resuspended in culture medium. JC-1 forms aggregates in normal mitochondria, emitting red fluorescence, whereas in depolarized mitochondria, it exists as monomers that generate green fluorescence [34]. The fluorescence intensity was observed under a fluorescence microscope (Olympus IX73, Japan) and quantified with a BD LSRFortessa flow cytometer (Becton Dickinson, Franklin Lakes, NJ, USA). The data were analyzed with FlowJo software, and the ΔΨm was expressed as the ratio of red to green fluorescence intensity.

### Reactive oxygen species generation

For the in vitro test, intracellular ROS levels were measured using DCFH-DA (2′,7′-dichlorodihydrofluorescein diacetate) reagent according to the manufacturer’s instructions. The suitably treated PC12 and SH-SY5Y cells were washed twice with PBS, and incubated with 10 µmol/L DCFH-DA fluorescent probe at 37 °C for 30 min. Fluorescence images were captured using a fluorescence microscope (Olympus IX73, Japan) and analyzed with ImageJ software.

### Apoptosis assay

The percentage of apoptotic rate of cells was determined via the terminal deoxynucleotidyl transferase mediated dUTP nick end labeling (TUNEL) assay, according to established protocols. The TUNEL assay was performed utilizing the TUNEL apoptosis detection kit, where 4’, 6-diamidino-2-phenylindole (DAPI) was used to stain the nuclei. TUNEL-positive cells and total cells were recorded via fluorescence microscopy (Olympus IX73, Japan).

### Real-Time quantitative polymerase chain reaction (RT–qPCR)

RT-qPCR was performed on RNA samples extracted from cultured cells in our study. Total RNA was extracted via TRIzol reagent after treatment. Briefly, the concentration of total RNA was quantified via a NanoDrop One spectrophotometer, and 1 µg of total RNA was used for reverse transcription. RT–qPCR analysis was performed using the First-Strand cDNA Synthesis Kit combined with the SYBR Green Premix Pro Taq HS qPCR kit (Accurate Biotechnology, Changsha, China) in accordance with the manufacturers’ protocols. The levels of target mRNAs were quantified by RT–qPCR using a LightCycler^®^480 II instrument (Roche, Switzerland). The primers designed and used for RT–qPCR are described in Supplementary Table 1.

### Western blot (WB)

Both cellular samples and spinal cord tissues were lysed with RIPA lysis buffer. Protein concentrations were measured using a BCA protein assay kit. Proteins were separated via 10% sodium dodecyl sulfate polyacrylamide gel electrophoresis (SDS–PAGE) and then transferred onto a nitrocellulose (NC) membrane (Millipore, USA). The membrane was blocked overnight at 4 °C with the appropriate primary antibodies. Next, the membrane was incubated for 1 h at room temperature with HRP-conjugated secondary antibodies (1:10000 dilution). The primary antibodies and dilutions used were as follows: HK2 (1:10000, Proteintech), glucose-6-phosphate isomerase (GPI, 1:300, Proteintech), enolase 2 (ENO2, 1:2000, Proteintech), triosephosphate isomerase 1 (TPI1, 1:5000, Proteintech), FOXK1 (1:1000, Boster), growth associated protein-43 (GAP43, 1:1000, Cell Signaling Technology), BCL-2 (1:1000, Proteintech), BAX (1:2000, Proteintech), and β-actin (1:10000, Proteintech). Western blot detection was performed using enhanced chemiluminescence (ECL) reagents. Finally, the optical densities were measured via a gel imaging system (Amersham ImageQuant 800, Cytiva, USA), and the data were analyzed via ImageJ software.

### Lactate assay

For the lactate assay, PC12 cells and SH-SY5Y cells from each group were pretreated with H_2_O_2_, 2-DG, or siFOXK1. The lactic acid content in supernatants was detected via a lactic acid colorimetric assay kit (Elabscience, Wuhan, China) following the manufacturer’s instructions. Briefly, 5 µL of each sample and 120 µL of lactic acid working solution were added to each well of a 96-well plate. The plate was incubated at 37 °C for 10 min, after which the termination solution was added to stop the reaction. The absorbance of each well at 530 nm was measured using a microplate reader. Results were normalized to the negative control (cell-free medium) and the experiment was performed in three independent replicates.

### Glycolysis analysis

A Seahorse XF96 Extracellular Flux Analyzer (Agilent Technologies, Santa Clara, CA, USA) was employed to conduct real-time analysis of glycolysis. One day prior to the assay, PC12 and SH-SY5Y cells were seeded in a Seahorse XF96 culture microplate (Agilent Technologies) at a seeding density of 4 × 10⁴ cells per well. Seahorse XF calibrant (Agilent Technologies) was loaded into the utility plate; subsequently, both the sensor cartridge and utility plate were incubated overnight in a CO₂-free incubator. On the day of the assay, cells were washed twice with complete Seahorse XF DMEM (supplemented with 10 mM glucose, 1 mM sodium pyruvate, and 2 mM glutamine), followed by equilibration in a CO₂-free incubator. For the glycolytic rate assay, cells were sequentially treated with 0.5 µM rotenone/antimycin A (Rot/AA) and 50 mM 2-DG. All assays were conducted in strict accordance with the manufacturer’s protocol, and the number of cells per well was normalized via direct cell counting to obtain absolute cell counts for data correction.

### Enzyme-linked immunosorbent assay (ELISA)

Using an ELISA kit, we analyzed the supernatants from PC12 and SH-SY5Y cells to determine the concentrations of TGF-β1 (ABclonal, Wuhan, China). Following centrifugation at 1000 g for 10 min, the supernatant was obtained. We strictly followed the instructions in the ELISA kit manual to measure the expression of TGF-β1. Optical density (OD) values were measured at 450 nm, and expression levels were calculated.

### Transfection with small interfering RNAs

Small interfering RNAs (siRNAs) were designed and synthesized by WZ Biosciences Inc. (Jinan, Shandong, China). For transfection mixture preparation, 5 µL of Lipo2000 (Invitrogen, USA) was diluted in 500 µL of Opti-MEM (Invitrogen, USA), while 5 µL of siRNA was diluted in another 500 µL of Opti-MEM. These two solutions were mixed, and the resulting mixture was incubated at 25 °C for 20 min prior to being added to a six-well plate pre-seeded with SH-SY5Y and PC12 cells. The knockdown efficiency of siRNA was evaluated using RT–qPCR and western blotting.

### Dual-luciferase reporter assay

293T cells were co-transfected with 250 ng of either HK2-WT plasmid, HK2-Mut plasmid or PGL3 reporter plasmid, together with 250 ng of FOXK1 expression plasmid or its corresponding negative control plasmid, respectively. An analysis of firefly luciferase activity versus Renilla luciferase activity was carried out by co-transfecting the cells with 50 ng Renilla plasmid. A dual luciferase reporter system (Yeasen Biotechnology, China) was used to measure luciferase activity 48 h after the transfection, following the manufacturer’s instructions. To assess the transcription efficiency of the reporter gene, firefly luciferase activity was normalized to that of Renilla luciferase.

### CUT&RUN assay

The CUT&RUN assay was performed on SH-SY5Y cells using the CUT&RUN Assay Kit (Vazyme, Nanjing, China). The antibody used in the CUT&RUN process was FOXK1 (Abcam, Cambridge, UK). This assay was conducted following the manufacturer’s protocol. The quantification of the purified DNA sample was conducted using the qRT-PCR method.

### Animals

All the animal experiments were conducted with the approval of the Jinan Central Hospital Experimental Animal Welfare Ethics Review Committee (approval number: JNCHIACUC2024-24). Female Wistar rats weighing 200–250 *g* were obtained from Jinan Pengyue Experimental Animal Technology Co., Ltd. and housed in the animal laboratory. The rats were allowed a one-week acclimatization period under a specific pathogen-free (SPF) barrier environment, with the following housing conditions: four rats per cage, ambient temperature maintained at 20–26 °C, a 12 h light-dark cycle, and a relative humidity ranging from 40% to 70%. All experimental procedures were performed in accordance with the ethical guidelines of the Experimental Animal Welfare Ethics Review Committee of Jinan Central Hospital and the Animal Research: Reporting of In Vivo Experiments (ARRIVE) guidelines.

### Establishment of the rat model of SCI

The SCI animal model was induced via a spinal cord impactor (RWD, Shenzhen, China) [35]. The rats were randomly divided into three groups: the Sham, SCI + PBS, and SCI + ADSCs groups. The rats were intraperitoneally injected with 3% pentobarbital sodium at a dose of 50 mg/kg for anesthesia. A midline incision was then made over the dorsal thoracic vertebrae of the rats, followed by a T9-T10 laminectomy to fully expose the spinal cord. A precision SCI impactor was used to strike the T10 spinal cord at a speed of 1.0 m/s, a depth of 2.0 mm, and a dwell time of 2.0 s. This resulted in rapid vascular congestion and tissue erythema in the T10 area. Transient twitching and convulsions of the tail and hind limbs verified the successful establishment of the SCI model. Hemostasis was then achieved, the wound was sutured, and iodine was applied to the sutured skin. For sham-operated rats, the spinal cords were exposed via the same procedure but without undergoing impact injury. In the ADSC-treated groups, a total of 1 × 10^6^ cells in 10 µL of PBS were administered via two injections applied 2 mm rostral and 2 mm caudal to the lesion via a microliter syringe.

### Intrathecal adenovirus injection

The experimental rats were randomly assigned to two groups: the shNC + SCI + ADSCs group and the shFOXK1 + SCI + ADSCs group. Prior to establishing the SCI model, the expression of FOXK1 in spinal cord neurons was inhibited via intrathecal injection of adeno-associated virus (AAV).

Rats were anesthetized via intraperitoneal injection of 3% sodium pentobarbital at a dose of 50 mg/kg, followed by viral suspension administration using the procedure described below: each rat was restrained with one hand to arch its back, followed by lumbar puncture via a microsyringe inserted into the intervertebral space between the L5 and L6 vertebrae. The syringe plunger was then gently pressed to deliver 10 µL of AAV viral suspension (WZ Biosciences Inc., China; serotype AAV9, titer 9.16 × 10¹³ vector genomes per mL (VG/mL)) to the AAV-shFOXK1 or AAV-shNC group. To confirm FOXK1 inhibition, FOXK1 protein expression was evaluated via Western blot and immunofluorescence assays at 21 days after injection. Four weeks later, rats in both groups underwent SCI induction following the previously described protocol.

### Locomotion recovery assay

To assess the locomotor ability of rats after SCI, the Basso–Beattie–Bresnahan (BBB) locomotion rating scale was employed. On post-surgery days 1, 3, 7, 14, 21, 28, 35, and 42, each rat was given 15 min of free movement in an open field. The BBB scale, ranging from 0 to 21 points, was used to assess hindlimb locomotion ability (with 0 indicating paralysis and 21 indicating normal mobility, assessing limb coordination and movement) [36].

An inclined plane assay was conducted to assess hindlimb strength by measuring the maximum angle at which rats could remain on the inclined surface for at least 5 s. Each mouse was positioned on the inclined board such that its body’s longitudinal axis aligned with that of the board, with the head facing the elevated end.

Six weeks after SCI, gait analysis was performed via the fully automated CatWalk XT system (version 10.6, Noldus) [37]. Rats received pre-training one week prior to the test to traverse the illuminated walkway. Between trials with individual rats, the track was thoroughly wiped with a 1% alcohol solution and dried to minimize odor interference. Experimental data were analyzed via the Catwalk XT system software.

### Histopathological staining and analysis

The rats were euthanized humanely on day 42 for evaluation of the inflammatory response, neuron regeneration, and remyelination of spinal cord. Fixed spinal cord tissues were dehydrated, wax immersed, embedded, and sectioned into 4 μm-thick slices. Hematoxylin and eosin (HE) staining was applied to the spinal cord tissues to evaluate their general histology and inflammatory cell infiltration. For assessment of neuronal morphology and density, tissue sections were subjected to toluidine blue-based Nissl staining. Luxol fast blue (LFB) staining was performed to determine the extent of myelin content. Additionally, the main organs including the heart, liver, spleen, lung, kidney, and brain after fixation were also collected and stained with HE to evaluate the in vivo safety of ADSCs. All histopathological changes and lesions were observed using an automatic digital slide scanner (Pannoramic MIDI, 3DHISTECH, Hungary).

Lesion areas were manually delineated using the “Freehand Selection” tool, with 3 animals per group included for statistical analysis.

For Nissl-stained sections, a total of 3 random non-overlapping fields were selected per section: 1 field in the lesion epicenter and 2 fields in the lesion edge; the “Cell Counter” plugin was then used to count intact Nissl-positive neurons, with only those displaying visible Nissl bodies and clear nuclear boundaries included.

For LFB-stained sections, 3 random non-overlapping fields were selected per section, covering the lesion epicenter and adjacent white matter tracts; the “Threshold” tool was used to delineate LFB-stained regions (myelinated tissue) for separating stained vs. unstained areas, and the LFB positive area percentage was calculated as (LFB-positive area / total ROI area) × 100.

### Immunofluorescence staining and analysis

First, the rats were perfused with PBS, followed by subsequent perfusion with a 4% formaldehyde solution. A 2 cm-long spinal cord segment centered at the injury site was further fixed in 4% formaldehyde at 4 °C for 24 h. Immunofluorescence staining was performed on paraffin-embedded spinal cord sections. Following deparaffinization, tissue sections were incubated with primary antibodies against MAP2 (1:200, Proteintech) and GFAP (1:400, Proteintech), followed by incubation with Alexa Fluor 594-conjugated goat anti-rabbit IgG and Alexa Fluor 488-conjugated goat anti-mouse IgG secondary antibodies (1:200, Abcam) diluted in PBS. Nuclei were visualized via DAPI. Labeled proteins and cellular structures were visualized via immunofluorescence imaging, which was conducted using a confocal laser scanning microscopy (Leica, Germany).

Quantitative analysis of MAP2: Three random non-overlapping fields were selected, covering both the injury site (lesion epicenter) and regions adjacent to the lesion core; the “Threshold” tool was applied to segment specific MAP2 fluorescence from the background, with the threshold value standardized across all images to ensure consistency, and the Integrated Density (sum of gray values of all thresholded pixels) was measured for each ROI before being normalized to the total area of the analyzed ROIs to obtain the normalized MAP2 fluorescence intensity.

### Statistical analysis

Statistical analyses were conducted using GraphPad Prism 9.5.0 (San Diego, CA, USA). The data were checked for normality using the Kolmogorov–Smirnov or Shapiro–Wilk test. Statistical analysis was performed via unpaired t-tests and one-way or two-way analysis of variance, followed by Bonferroni’s post hoc test to determine significant differences between groups. A P-value less than 0.05 was considered statistically significant. The data are presented as the means ± standard deviations.

## Results

### Protective effect of ADSCs on nerve cells

**Fig. 1:**
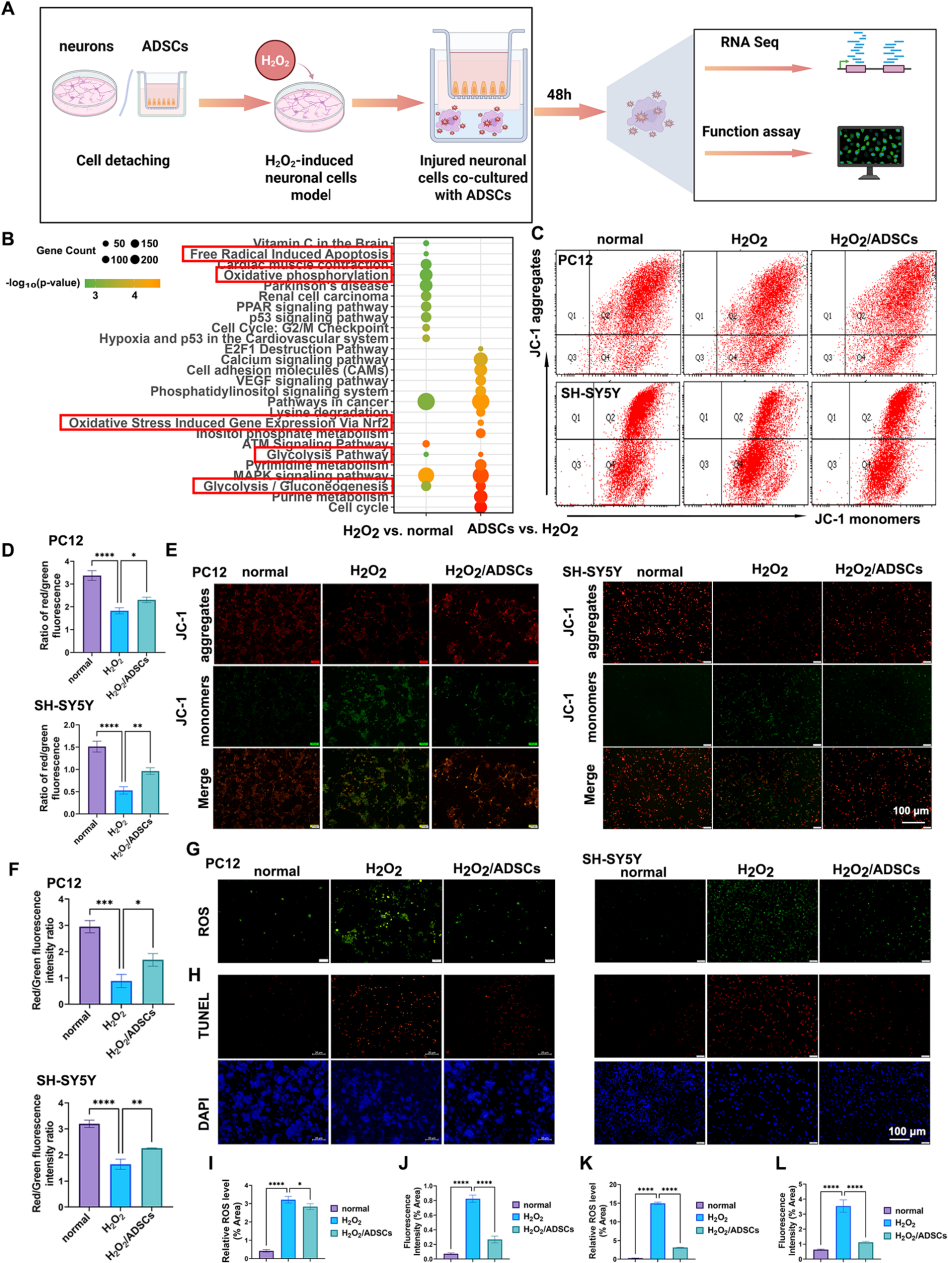
Protective effect of ADSCs on PC12 and SH-SY5Y cells. **A** Schematic illustration depicting the repair of H₂O₂-induced neuronal cells by ADSCs. **B** KEGG analysis revealed that significant changes occurred in oxidative stress and glycolytic metabolism. **C**–**D** Flow cytometry analysis of the mitochondrial membrane potential in PC12 and SH-SY5Y cells probed with JC-1. **E** Fluorescence analysis of the mitochondrial membrane potential in injured PC12 and SH-SY5Y cells. JC-1 aggregates are shown in red, and JC-1 monomers are shown in green. Scale bar, 100 μm. **F** Quantitative analysis of JC-1 aggregates and JC-1 monomers fluorescence intensity. **G** Fluorescence microscopy revealed that the fluorescence intensity of ROS in ADSCs-treated group was significantly reduced. Scale bar, 100 μm. **H** Detection of cell apoptosis via the TUNEL assay. **I**–**L** Quantitative analysis of ROS and TUNEL assays. * is *P* < 0.05, **P < 0.01, *** is *P* < 0.001, **** is *P* < 0.0001 according to the one-way ANOVA

PC12 cells and SH-SY5Y cells exhibited oxidative stress, mitochondrial damage, and apoptosis when stimulated with H_2_O_2_ similar to the conditions observed in spinal cord cells of individuals with SCI. We determined the optimal working concentration of H_2_O_2_ by detecting reactive oxygen species (ROS), measuring mitochondrial membrane potential, and performing CCK-8 assays (Supplementary Fig. 1). To investigate the impact of ADSCs on nerve cell activity following H₂O₂ treatment, ADSCs were co-cultured with PC12 and SH-SY5Y cells (Fig. 1A). Considering the above experimental results, transcriptomic sequencing analysis was performed on PC12 cells from the three groups (normal, H₂O₂, and H₂O₂ + ADSCs groups). The enriched KEGG pathways included those related to oxidative stress. Additionally, the glycolysis pathway exhibited obvious changes before and after co-culture with ADSCs (Fig. 1B).

The results showed that ADSCs could increase the mitochondrial membrane potential, reduce the production of ROS, and inhibit the apoptosis of PC12 and SH-SY5Y cells. First, we evaluated the mitochondrial activity and functions of repaired neuronal cells treated with ADSCs. The mitochondrial membrane potential, a global indicator of the state and function of healthy mitochondria, was assessed via JC-1 dye. The ratio of JC-1 aggregates to JC-1 monomers is commonly used to assess mitochondrial membrane potential. As shown in Fig. 1C and D, the ratio of red/green fluorescence intensity in injured PC12 and SH-SY5Y cells markedly increased after ADSCs treatment. Furthermore, the JC-1 images revealed that the red fluorescence of JC-1 aggregates (indicating healthy mitochondria) in the repaired neuronal cells treated with ADSCs was significantly increased, whereas the green fluorescence of JC-1 monomers (indicating unhealthy mitochondria) was decreased (Fig. 1E, F). Additionally, due to the recovery of mitochondrial function in damaged neuronal cells, ADSCs co-culture reduced ROS levels in PC12 and SH-SY5Y cells (Fig. 1G, I, K). The results obtained via the use of the DCFH-DA probe revealed that H_2_O_2_ stimulation significantly increased the fluorescence intensity of intracellular ROS compared with that in control group. However, treatment with ADSCs resulted in a significant reduction in ROS fluorescence intensity following H_2_O_2_ stimulation. TUNEL assays revealed increased apoptosis in the H_2_O_2_-treated group and decreased apoptosis in the ADSCs group (Fig. 1H, J, L).

### ADSCs improved motor function and optimized pathology in SCI rats

Initially, we evaluated the potential risks associated with the use of ADSCs following SCI. The hearts, livers, spleens, lungs, kidneys, and brains of rats were examined via HE staining to evaluate ADSCs effects on day 42 post-surgery. The results confirmed that local injection of ADSCs did not cause organ damage in rats (Supplementary Fig. 2).

**Fig. 2:**
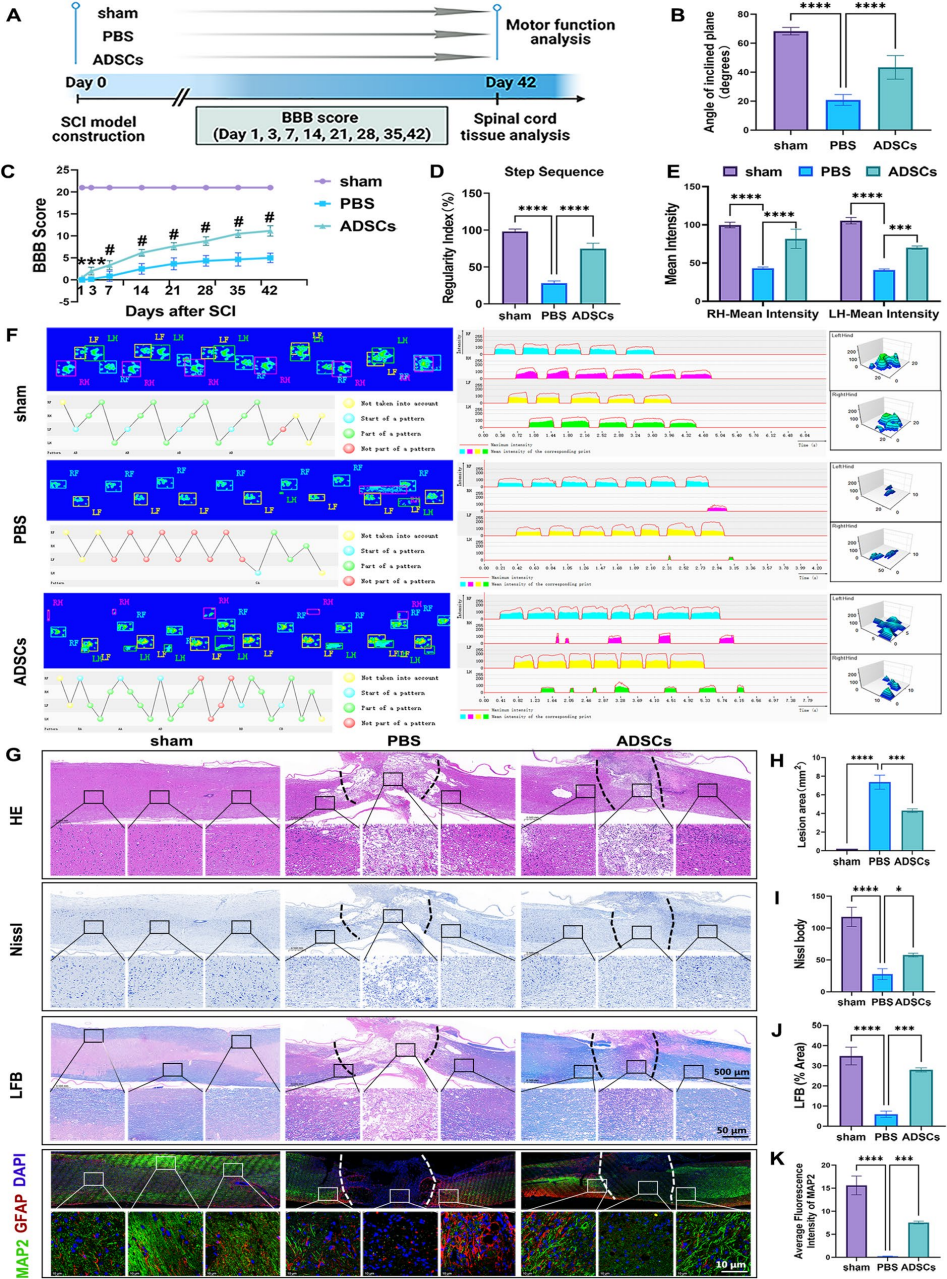
ADSCs improved motor function and optimized pathology in SCI rats. **A** Schematic of the SCI model construction, the migration of PBS and ADSCs to the injured spinal cord, and the experimental schedule for functional analysis. **B** Quantitative analysis of the maximum angle at which the rats maintained their position on the inclined plane for 5 s to assess hindlimb strength (*n* = 6; **** *P* < 0.0001, one-way ANOVA test, Tukey’s multiple comparisons test). **C** BBB scores at 6 consecutive weeks after SCI (*n* = 6, one-way ANOVA test, Tukey’s multiple comparisons test; *** *P* < 0.001, # *P* < 0.0001, the comparison of the PBS and ADSCs groups). **D**–**E** Quantification of the step sequence and mean intensity of the hind limbs movement at 6 weeks post-injury (*n* = 3; *** *P* < 0.001, **** *P* < 0.0001, one-way or two-way ANOVA tests, Tukey’s multiple comparisons test). **F** Representative footprints used to analyze the recovery of hindlimb motor function. **G**–**K** Longitudinal spinal cord sections obtained from the groups on day 42 after SCI were examined via HE staining, Nissl staining, and LFB staining. The inset images are magnified in the lower lane (above, scale bar, 500 μm; below, scale bar, 50 μm). Immunofluorescence analysis of neuronal markers at week six after SCI. MAP2 (green) and GFAP (red); scale bar = 10 μm. All the data are presented as the means ± SDs (*n* = 3). *P < 0.05, *** *P* < 0.001, **** *P* < 0.0001. Significance was calculated via one-way ANOVA followed by Tukey’s multiple comparisons test

Considering that ADSCs transplantation induces neuronal repair and remyelination in injured spinal cords, we anticipate that this strategy may restore locomotor function following SCI. To detect the effect of ADSCs on the motor function of rats with SCI, we used inclined plane tests, BBB scores, and CatWalk analysis to evaluate the motor function of mouse hind limbs (Fig. 2A). An inclined plane test was performed to evaluate hindlimb strength by determining the maximum angle at which the mouse stayed on the inclined plane for at least 5 s [38]. Compared with PBS-treated SCI rats, ADSCs-treated rats maintained a significantly larger angle on the inclined planes, which indicates good recovery of hindlimb strength (Fig. 2B). Locomotor function recovery was comprehensively evaluated via the BBB scoring system in a double-blinded manner. Remarkably, treatment with ADSCs significantly improved hindlimb locomotor function in SCI rats, with intermittent palmar bearing support and palmar bearing movement observed at 6 weeks post-injury (Fig. 2C). Compared with that of the PBS group, the performance of the ADSCs treatment group was significantly greater in several CatWalk analysis measures, such as step sequence, mean intensity, and print view (Fig. 2D-F).

Finally, all the rats were sacrificed at 6 weeks after ADSCs treatment. HE staining revealed the morphological changes in the spinal cord tissue following surgery, as illustrated in Fig. 2G. The spinal cord tissue in the sham group exhibited a distinct and compact structure with a typical neuronal configuration. In contrast, the PBS group presented pronounced damage, structural disorganization and substantial cavitation, characterized by extensive vacuolar alterations. Notably, ADSCs promoted the repair of spinal cord tissue damage, and facilitated the restoration of the cavity (Fig. 2H). In addition, to understand the extent of neuronal damage, Nissl staining was also performed. This revealed cytoplasmic shrinkage and nuclear condensation of the axonal neurons at the injury site in the PBS group. In contrast, ADSCs helped attenuate these histopathological changes (Fig. 2G, I). The results from LFB staining confirmed that ADSCs promote myelin regeneration in the injured spinal cord. Specifically, LFB staining, which selectively labels myelin lipids, significantly increased the intensity and area of myelin staining at the lesion site in the ADSCs-treated groups (Fig. 2G, J). Finally, the neuronal cells and astrocytes were labeled via immunofluorescence staining. The results revealed that ADSCs treatment significantly promoted neuronal cell survival after SCI (Fig. 2G, K). GFAP-positive astrocytes exhibit increased activation (enhanced fluorescence intensity) in the injured spinal cord compared to the sham group. ADSCs transplantation significantly reduced GFAP fluorescence intensity (Fig. 2G). Overall, our research confirmed that ADSCs treatment has beneficial therapeutic effects, promoting injured spinal cord repair and enhancing functional recovery in rats.

### ADSCs promote the repair of damaged nerve cells by regulating glycolytic metabolism

**Fig. 3:**
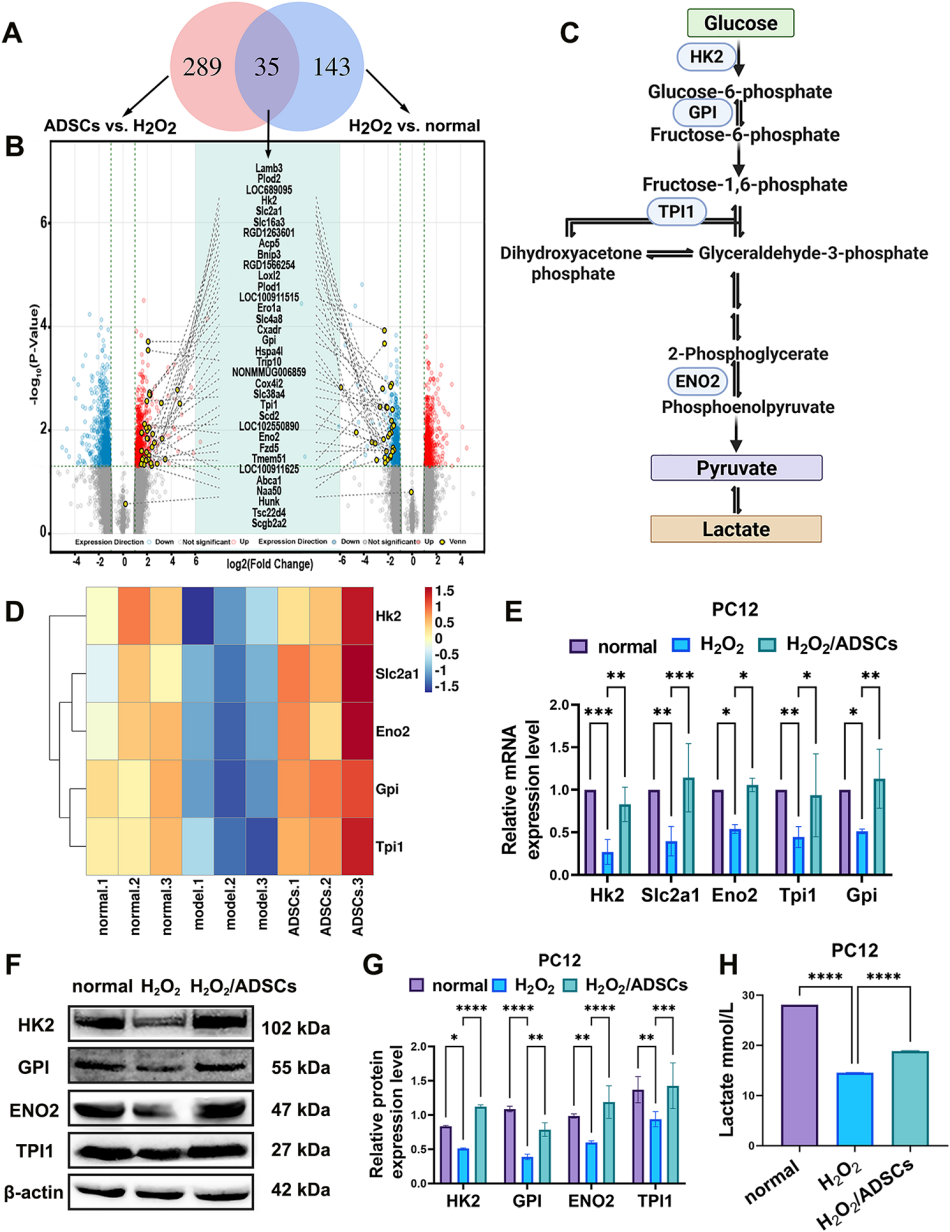
ADSCs promote the repair of damaged PC12 cells by regulating glycolytic metabolism. **A** Venn diagram analysis was used to identify the intersection of DEGs from H₂O₂-treated damaged PC12 cells and those from damaged PC12 cells co-cultured with ADSCs, on the basis of transcriptomic data. **B** Volcano plot analysis of DEGs in the two compared groups. Red represents upregulated genes, and blue represents downregulated genes (*n* = 3). **C** Schematic diagram of glycolytic metabolism. **D** Heatmap of the transcriptome sequencing results. Among these DEGs, multiple key genes involved in glycolytic metabolism, including Hk2, Slc2a1, Eno2, Gpi, and Tpi1 were identified. **E** RT–qPCR detection of the expression of glycolytic genes. **F**–**G** Detection of the expression of glycolysis-related proteins via Western blotting. **H** Detection of lactic acid in the supernatant. *P < 0.05, **P < 0.01, *** *P* < 0.001, **** *P* < 0.0001; one-way or two-way ANOVA tests, Tukey’s multiple comparisons test

To explore the molecular basis underlying ADSCs-mediated neuroprotection, transcriptome sequencing was performed to identify differentially expressed genes (DEGs) between damaged PC12 cells (treated with H₂O₂) and those co-cultured with ADSCs. Venn diagram and volcano plot analyses of the DEGs were performed. In total, 467 differentially expressed genes were identified. Multiple key genes involved in glycolysis, such as Hk2, Slc2a1, Eno2, Gpi and Tpi1, presented distinct expression patterns (Fig. 3A and D). To validate these sequencing results, we performed further analyses on PC12 cells, confirming the altered expression of these genes at both mRNA and protein levels. Specifically, significant downregulation of glycolysis-related genes was observed in the H_2_O_2_-treated group, whereas marked upregulation of these genes was detected in damaged PC12 cells treated with ADSCs (Fig. 3E and G). The lactate concentration, an important indicator of glycolytic activity, also changed significantly among the groups (Fig. 3H). By combining the results of gene expression profiling, protein level analysis and metabolite measurement, we observed notable alterations in glycolytic metabolism. These findings indicate that ADSCs may repair damaged PC12 cells by increasing glycolytic metabolism.

### Inhibition of glycolytic metabolism attenuates the functional recovery of ADSC-treated neurons

**Fig. 4:**
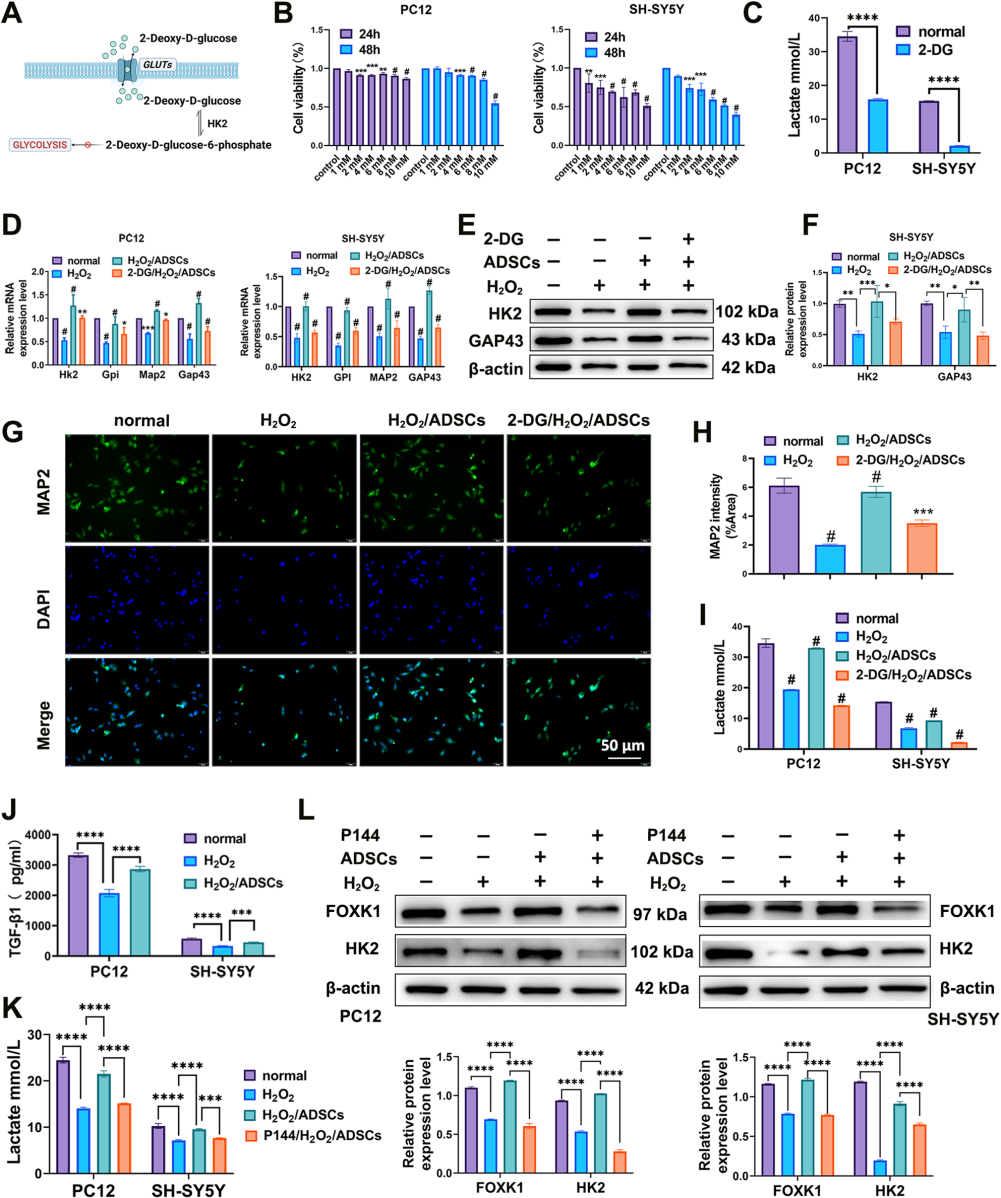
Inhibition of glycolytic metabolism attenuates the nerve injury repair function of ADSCs. **A** Schematic diagram of the inhibition of glycolysis by 2-DG. **B** A CCK-8 assay was used to detect the viability of PC12 and SH-SY5Y cells after treatment with different concentrations of 2-DG. **C** Detection of lactic acid. **D** Detection of glycolysis-related mRNA expression via RT–qPCR. **E**–**F** Detection of glycolysis-related protein expression in SH-SY5Y cells via Western blotting. **G**–**H** Immunofluorescence was used to detect the expression of MAP2 in SH-SY5Y cells. **I** Detection of lactic acid in the supernatant. **J** Quantification of TGF-β1 secretion in cell culture supernatants by ELISA. **K** Detection of lactic acid in the supernatant. **L** Detection of glycolysis-related protein expression in PC12 and SH-SY5Y cells via Western blotting.*P < 0.05, **P < 0.01, *** *P* < 0.001, **** *P* < 0.0001, # *P* < 0.0001; one-way or two-way ANOVA tests, Tukey’s multiple comparisons test

2-DG is a glucose analog that acts as a competitive inhibitor of glycolysis, primarily by targeting the early steps of the pathway to block glucose metabolism [39]. We utilized the glycolytic inhibitor 2-DG to evaluate the involvement of glycolytic metabolism in the neurorepair process mediated by ADSCs (Fig. 4A). Cell viability and lactate concentrations were detected after treatment with 2-DG. The results of the CCK-8 assay revealed that the viability of PC12 cells treated with 10 mM 2-DG for 48 h and SH-SY5Y cells treated with 8 mM 2-DG for 48 h decreased to 50%. Therefore, 10 mM and 8 mM were determined to be the optimal concentrations of 2-DG for PC12 and SH-SY5Y cells, respectively (Fig. 4B). The expression of lactic acid in the supernatant revealed that 2-DG inhibited the production of lactic acid (Fig. 4C). Neuronal and glycolysis markers were detected via RT–qPCR and western blotting after treatment with different combinations of H_2_O_2_, ADSCs, and 2-DG. The results showed that, in H_2_O_2_-induced neurons, the expression levels of HK2, GPI, MAP2, and GAP43 decreased significantly. However when the cells were treated with ADSCs, this decrease was reversed. Compared with those in the H_2_O_2_ + ADSCs group, HK2, GPI, MAP2, and GAP43 expression levels were lower in the 2-DG +H_2_O_2_ + ADSCs group (Fig. 4D and F). Immunofluorescence staining revealed that the neuroprotective effect of ADSCs was attenuated by 2-DG (Fig. 4G, H). Lactate detection analysis revealed that lactate secretion was significantly lower in the 2-DG +H_2_O_2_ + ADSCs group than in the H_2_O_2_ + ADSCs group (Fig. 4I). These findings demonstrate that inhibition of glycolytic metabolism attenuates the ability of ADSCs to repair nerve injury.

ADSCs-conditioned medium (CM) experiments provide definitive evidence that ADSCs exert their effects via paracrine secretion (Supplementary Fig. 3). ELISA confirmed significantly elevated TGF-β1 levels in the supernatant of ADSC-injured neuron co-cultures, verifying paracrine secretion of TGF-β1 by ADSCs (Fig. 4J).​ Treatment with Disitertide diammonium (P144), a specific peptide inhibitor of TGF-β1-receptor binding, at a concentration of 100 µg/ml in the co-culture system resulted in decreased lactate secretion and reduced neuronal FOXK1 and HK2 protein expression compared with the untreated co-culture group (Fig. 4K, L). These data directly demonstrate that blocking TGF-β1 signaling abolishes ADSC-induced activation of the FOXK1-HK2 axis and subsequent glycolytic enhancement, confirming TGF-β1 as a critical paracrine mediator.​.

### ADSCs enhance glycolysis and neuroprotection in injured neural cells via the FOXK1/HK2 pathway

The transcription factor FOXK1 plays a pivotal role in regulating glycolytic metabolism, exerting a significant influence on a series of rate-limiting enzymes in glycolysis. On the basis of the above experimental results, we hypothesized that ADSCs promote the repair of injured nerve cells by regulating glycolytic metabolism through the FOXK1/HK2 pathway. To investigate this hypothesis, we examined the effect of knocking down FOXK1 on HK2 expression in PC12 and SH-SY5Y cells.

**Fig. 5:**
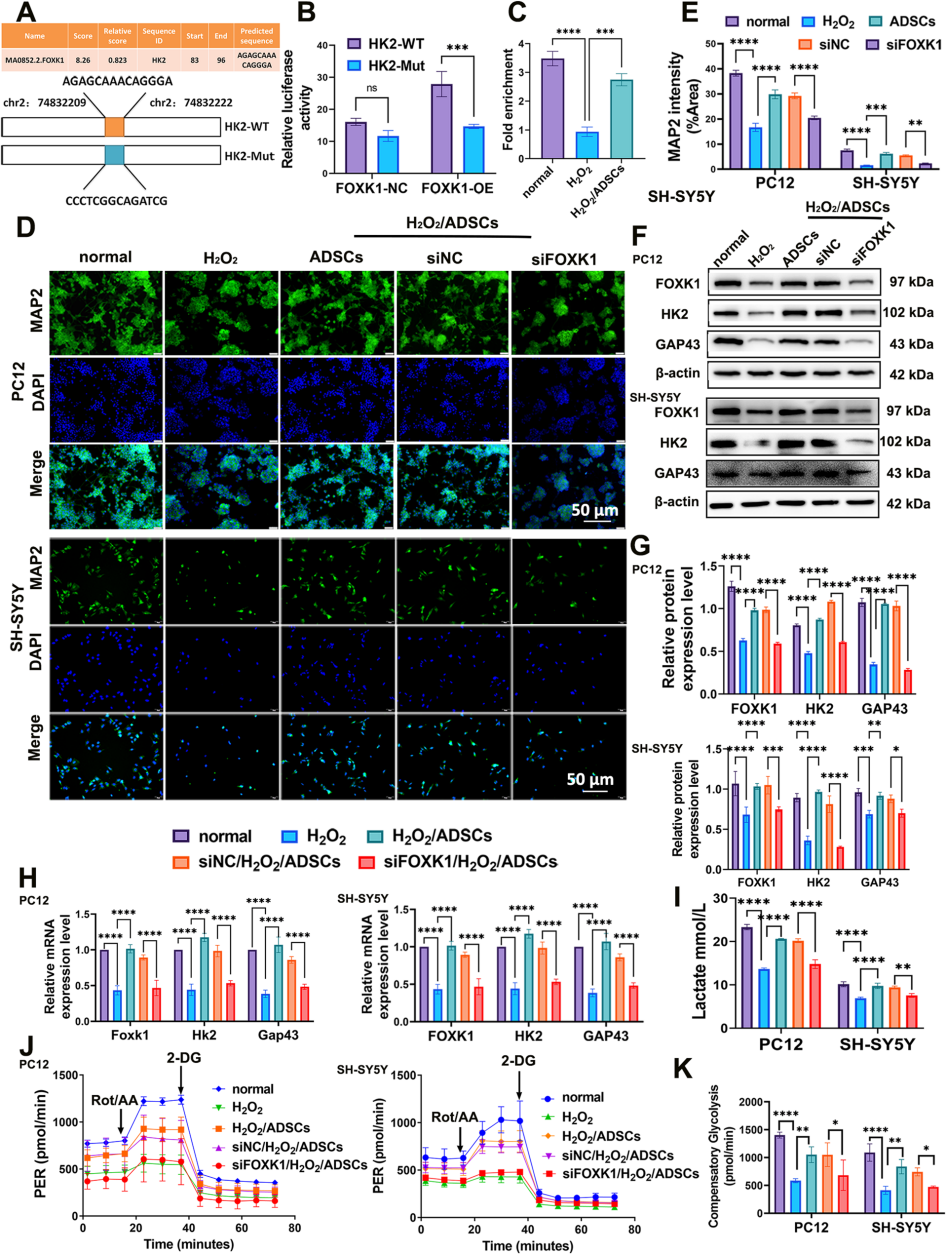
ADSCs enhance glycolysis and neuroprotection in injured neural cells via FOXK1/HK2 pathway. **A** Schematic representation of the predicted binding sites of FOXK1 in the promoters of HK2 and matched mutant sequences. **B** Dual luciferase reporter assay showed the luciferase activity of FOXK1 on the wild-type and mutant promoters of HK2. **C** Cut&Run assay provided direct evidence that FOXK1 binds to the *HK2* promoter. **D**–**E** Immunofluorescence was used to detect the expression of MAP2 in PC12 and SH-SY5Y cells. **F**–**H** The protein and mRNA expression levels of FOXK1, HK2, and GAP43. **I** Detection of lactate in the supernatant. **J** A representative Seahorse glycolytic rate assay was used to assess cellular glycolytic metabolism. **K** Calculated glycolytic proton efflux rate (glycoPER) for compensatory glycolysis. *P < 0.05, **P < 0.01, *** *P* < 0.001, **** *P* < 0.0001; one-way or two-way ANOVA tests, Tukey’s multiple comparisons test

To further validate that FOXK1 directly influences the transcription of HK2, we analyzed of the binding sites and motifs of FOXK1 in its promoter. We constructed wild-type and mutant plasmids with a luciferase reporter gene in the HK2 promoter region (Fig. 5A). The dual-luciferase reporter assay demonstrated that FOXK1 increased luciferase activity at the wild-type HK2 promoter, whereas luciferase activity was significantly reduced when the binding sites were mutated (Fig. 5B). Cut&Run assay provided direct evidence of FOXK1 binding to the HK2 promoter (Fig. 5C). These findings indicated that FOXK1 upregulated the expression of HK2 by activating its promoter.

We next explored whether inhibiting the expression of FOXK1 in PC12 and SH-SY5Y cells could weaken the neuroprotective effect of ADSCs. The immunofluorescence results revealed a significant reduction in the fluorescence intensity of MAP2 in the siFOXK1 + H₂O₂+ADSCs group (Fig. 5D, E). Compared with the siNC + H₂O₂+ADSCs group, the siFOXK1 + H₂O₂+ADSCs group exhibited reduced mRNA and protein expression levels of HK2, along with decreased expression of nerve cell-related markers GAP43 (Fig. 5F-H). These findings collectively suggest that the inhibition of FOXK1 in PC12 and SH-SY5Y cells impairs the ability of ADSCs to upregulate HK2 expression, which is consistent with the previously proposed regulatory relationship between FOXK1 and HK2 in glycolytic metabolism. More importantly, the downregulation of GAP43 and MAP2, directly reflects that the neuroprotective and regenerative effects of ADSCs are attenuated when FOXK1 is silenced.

Lactate, the end product of glycolysis, directly reflects the flux of the glycolytic pathway. Compared with those in the siNC + H₂O₂+ADSCs group, the lactate production in the siFOXK1 + H₂O₂+ADSCs group was significantly lower (Fig. 5I), which indicates that silencing FOXK1 impairs the ability of ADSCs to increase glycolytic output in injured PC12 and SH-SY5Y cells. We performed a Seahorse glycolytic rate assay to determine whether co-culturing ADSCs with neural cells with inhibited FOXK1 expression altered neural cell glycolytic metabolism. Following H₂O₂-induced injury, the glycolytic metabolism of PC12 and SH-SY5Y cells was reduced; however, co-culture with ADSCs promoted glycolytic metabolism in these neural cells. Notably, after FOXK1 expression was inhibited in neural cells, the pro-glycolytic metabolic effect of ADSCs on injured neural cells was also suppressed (Fig. 5J), leading to decreased compensatory glycolysis (Fig. 5K). Bioenergetics experiments revealed that glycolysis was induced by co-culture with ADSCs and suppressed by FOXK1 knockdown.

### Inhibiting FOXK1 impairs ADSCs’ neuroprotective effects and bladder functional recovery in SCI Rats

**Fig. 6:**
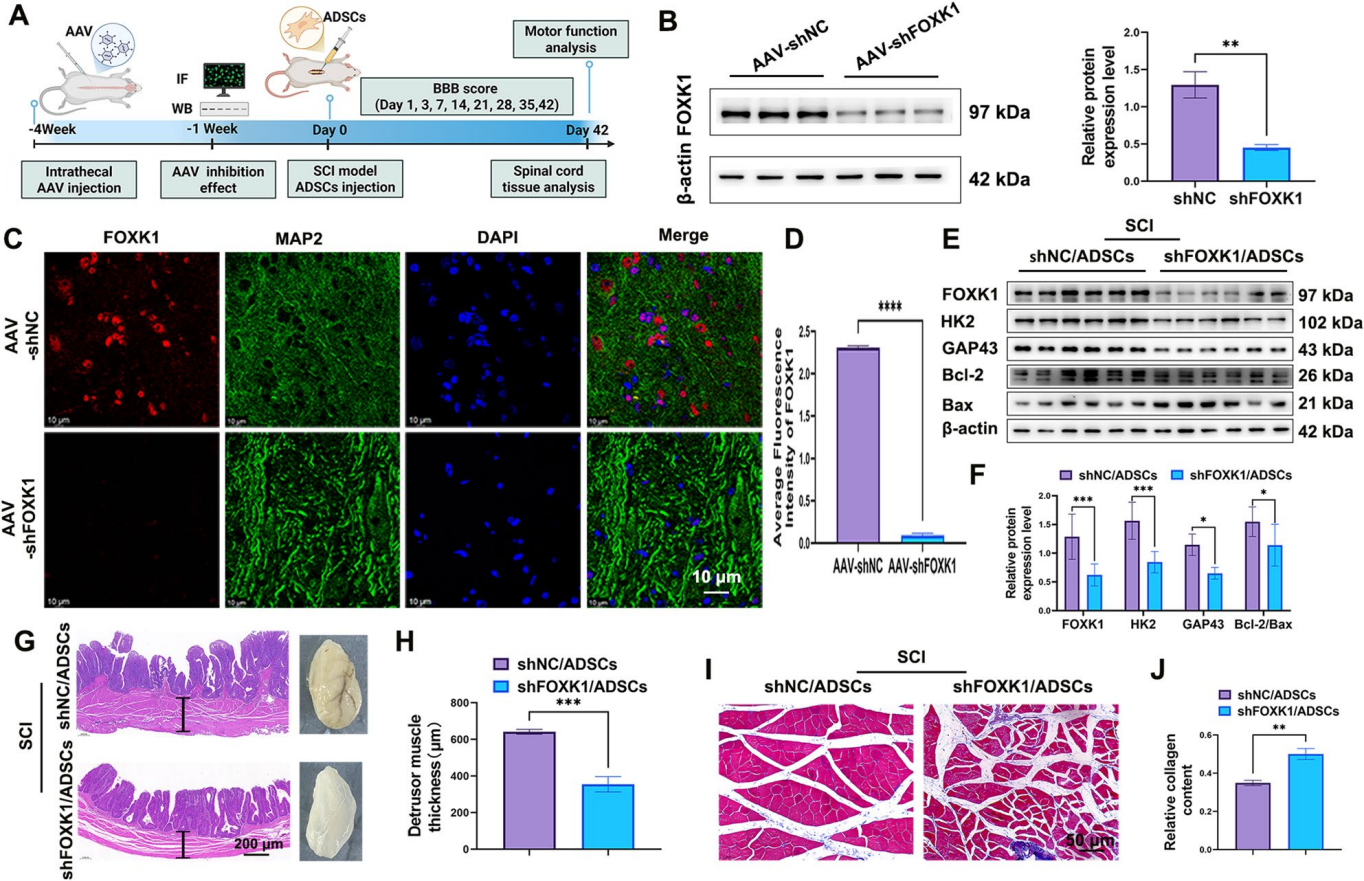
Inhibiting FOXK1 impairs the neuroprotective effects and bladder functional recovery of ADSCs in SCI rats. **A** Timeline of treatment regimens for SCI rats in the SCI + shNC + ADSCs and SCI + shFOXK1 + ADSCs groups. **B**–**D** AAV-shFOXK1 was injected into rats to suppress FOXK1 expression in neural cells. **E**–**F** Detection of protein expression via Western blotting. **G**–**H** Morphology and HE staining of the bladders of treated rats 42 days post-SCI are shown, with the detrusor muscle thickness marked by the black line (*n* = 3). Scale bar: 200 μm. **I**–**J** Masson staining of the gastrocnemius muscle in treated rats 42 days post-SCI. Scale bar: 50 μm. *P < 0.05, **P < 0.01, *** *P* < 0.001, **** *P* < 0.0001; one-way or two-way ANOVA tests, Tukey’s multiple comparisons test

To determine whether ADSCs facilitate recovery from spinal cord injury by regulating neuronal glycolytic metabolism via FOXK1, we conducted in vivo experiments in rats (Fig. 6A). Specifically, an adeno-associated virus (AAV) carrying short hairpin RNA targeting FOXK1 (AAV-shFOXK1) was injected into rats to suppress FOXK1 expression in neural cells. Subsequently, molecular biological assays and behavioral tests were conducted to systematically investigate the regulatory role of the ADSCs/FOXK1/glycolysis axis in SCI repair.

Immunofluorescence and Western blot assays demonstrated that the expression of FOXK1 in neural cells was significantly suppressed following AAV injection (Fig. 6B-D). We verified the neuroprotective effects of ADSCs in vivo via western blot analysis, which revealed significantly decreased levels of FOXK1 and HK2 in the SCI + shFOXK1 + ADSCs group (Fig. 6E, F). Furthermore, the expression of GAP43 was also significantly lower than that in the shNC group. Additionally, there were decreased protein expression levels of the anti-apoptotic marker BCL-2, and significantly up-regulated expression level of pro-apoptotic marker BAX in the SCI + shFOXK1 + ADSCs group. The above results indicate that the neuroprotective effect of ADSCs is attenuated after inhibition of neuronal FOXK1 expression in neurons.

Furthermore, the impact of ADSCs on bladder function was examined. As a result of bladder dysfunction resulting from nerve injury, the detrusor muscle layer typically experiences atrophy or alterations to muscle fibers, which ultimately results in a reduction in muscle thickness. Analysis of HE-stained sections from SCI mouse bladders demonstrated that SCI + shFOXK1 + ADSCs treatment led to a marked reduction in detrusor muscle layer thickness within the neurogenic bladder, indicating that nerve innervation restoration in the bladder was restricted (Fig. 6G, H). Masson staining results revealed that in the SCI + shFOXK1 + ADSCs group, the rats exhibited gastrocnemius muscle atrophy accompanied by an increase in muscle fiber content (Fig. 6I, J). These findings further emphasize the considerable beneficial impact of ADSCs on functional recovery following SCI, whereas the protective effects of ADSCs are attenuated when FOXK1 expression is suppressed.

### FOXK1 knockdown attenuates ADSCs-mediated functional recovery and neuroprotection in SCI Rats

**Fig. 7:**
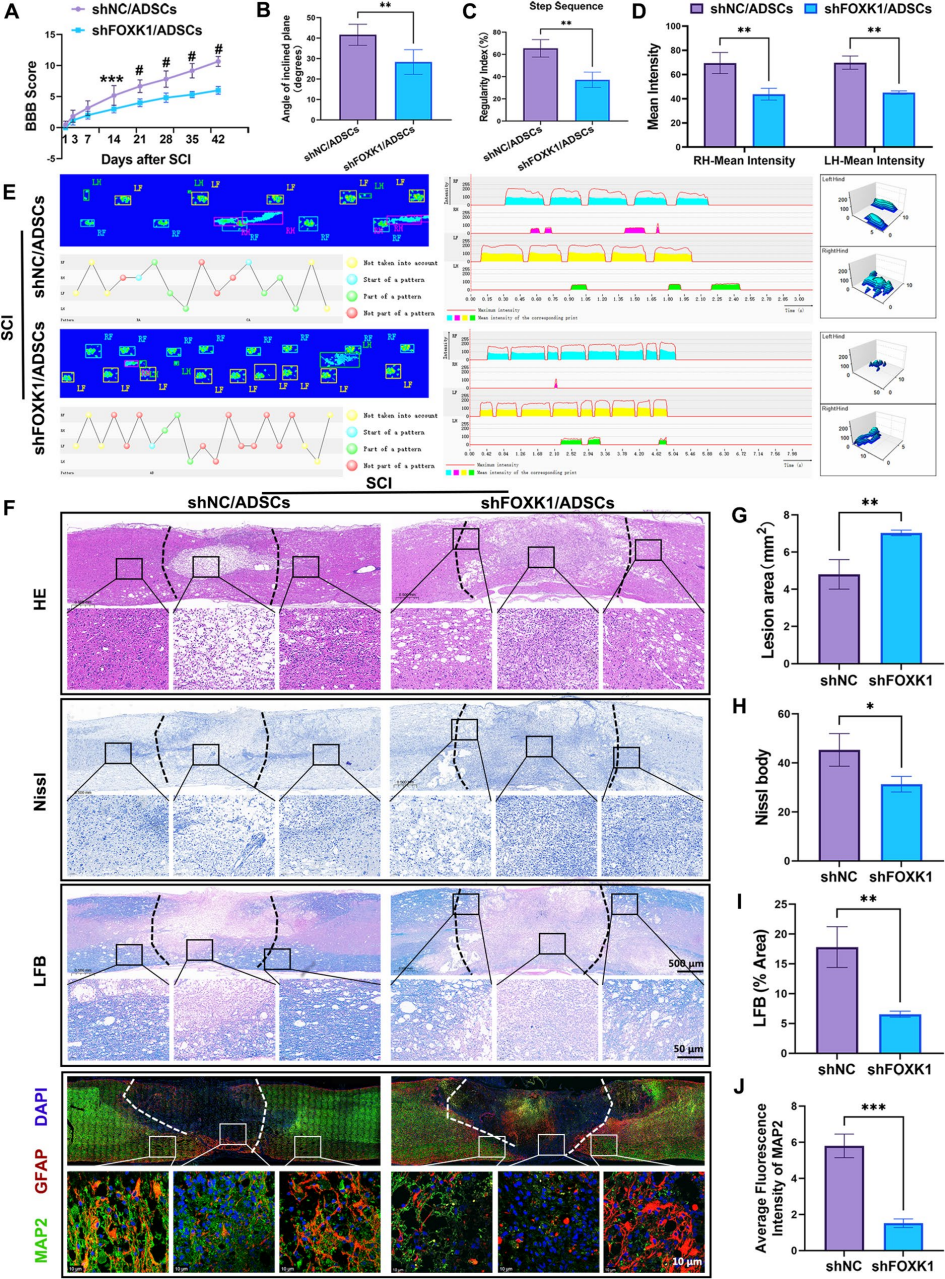
FOXK1 knockdown attenuates ADSCs-mediated functional recovery and neuroprotection in SCI rats. **A** BBB scores at 6 consecutive weeks after SCI. **B** Quantitative analysis of the maximum angle at which the rats maintained their position on the inclined plane for 5 s to assess hindlimb strength (*n* = 6; ** *P* < 0.01, one-way ANOVA test, Tukey’s multiple comparisons test). **C**–**D** Quantification of the step sequence and mean intensity of the hind limb movement at 6 weeks post-injury (*n* = 3; ** *P* < 0.01, one-way or two-way ANOVA tests, Tukey’s multiple comparisons test). **E** Representative footprints used to analyze the recovery of hindlimb motor function. **F**–**J** Longitudinal spinal cord sections obtained from the groups on day 42 after SCI were examined via HE staining, Nissl staining, and LFB staining. The inset images are magnified in the lower lane (above, scale bar, 500 μm; below, scale bar, 50 μm). Immunofluorescence analysis of neuronal markers at week six after SCI. MAP2 (green) and GFAP (red); scale bar = 10 μm. All the data are presented as the means ± SDs (*n* = 3). *P < 0.05, ** *P* < 0.01, *** *P* < 0.001. Significance was calculated via one-way ANOVA followed by Tukey’s multiple comparisons test

Through motor function assessments and pathological examinations, we further verified that ADSCs exert neuroprotective effects via FOXK1. SCI rats treated with shFOXK1 + ADSCs presented significant reductions in BBB scores, with significant differences observed after 14 days (Fig. 7A). Next, hindlimb strength and movement velocity were tested to further support the recovery of locomotor function following SCI with shFOXK1 + ADSCs treatment. Compared with shNC + ADSCs-treated SCI rats, shFOXK1 + ADSCs-treated SCI rats presented a significantly decreased angle on inclined planes, indicating poor recovery of hindlimb strength (Fig. 7B). These findings were further confirmed by the results of footprint analysis (Fig. 7C-E). The shFOXK1 + ADSCs group exhibited abnormal paw prints with decreased step sequence, mean intensity, and reduced interlimb coordination. These gait abnormalities correlated with the BBB scores, suggesting that FOXK1-mediated glycolysis influences not only neural cell survival but also the remodeling of neural circuits for locomotor function.

In the shFOXK1 + ADSCs group, HE sections revealed a larger cavitation area at the injury site compared to shNC + ADSCs group (Fig. 7F, G). This enlarged cavity was indicative of increased tissue loss and impaired repair, which is consistent with the observed behavioral deficits. Nissl staining further underscored the impact of FOXK1 knockdown on neural integrity. The shFOXK1 + ADSCs group showed a significant reduction in the number of intact Nissl bodies (Fig. 7F, H). Additionally, surviving neurons exhibited signs of degeneration, such as shrunken cell bodies and condensed chromatin, indicating that FOXK1 was essential for maintaining neuronal viability in the post-SCI environment.​ LFB staining confirmed that FOXK1 promoted myelin regeneration in the injured spinal cord (Fig. 7F, I). Immunofluorescence staining for MAP2, a dendritic marker, provided high-resolution insights into neuronal connectivity. Compared with those in the control group, the MAP2-positive dendrites in the shFOXK1 + ADSCs group exhibited a sparse and fragmented morphology at the injury border, accompanied by a significant reduction in fluorescence intensity (Fig. 7F, J).

Mechanistically, these histological changes aligned with the reduced HK2 expression and glycolytic activity in the shFOXK1 + ADSCs group. The energy deficit caused by FOXK1 knockdown likely impaired the synthesis of essential biomolecules required for neuronal survival, axonal growth, and glial scar organization. By disrupting the FOXK1-HK2 axis, we effectively abrogated the beneficial effects typically conferred by ADSCs, reinforcing the central role of this molecular pathway in mediating the therapeutic effects of stem cell transplantation.

## Discussion

In the present study, we conducted a systematic investigation and demonstrated that ADSCs exerted neuroprotective effects and facilitated injured spinal cord repair by enhancing glycolytic metabolism in damaged neurons. This process is mediated through the FOXK1-HK2 signaling axis. Vascular damage induced by spinal cord injury results in ischemia and hypoxia at the site of injury, and this pathological state further results in significant metabolic disruptions and neuronal apoptosis [40]. Previous studies have demonstrated that enhancing the energy supply of neurons can effectively decrease pathological death [41, 42]. Our findings provide new insights into the metabolic reprogramming of damaged neurons underlying stem cell-based therapy for SCI and identify a potential therapeutic target for improving functional recovery.

Neurons obtain ATP from the tricarboxylic acid (TCA) cycle and oxidative phosphorylation (OXPHOS) [43, 44]. The ischemic-hypoxic microenvironment post-SCI results in metabolic disorders that include disturbances in glucose metabolism, lipid metabolism, and mitochondrial dysfunction [45]. Our results revealed that mitochondrial function was significantly reduced in H₂O₂-damaged neuronal cells. ADSCs improved the mitochondrial membrane potential, inhibited neuronal oxidative stress, and promoted neuronal metabolic reprogramming. Energy metabolism disorder is a central hallmark of neuronal damage post-SCI [46]. Insufficient ATP production disrupts neuronal homeostasis and triggers apoptosis [47]. This energetic crisis forces neurons to rely on alternative metabolic pathways to sustain ATP production and survival. Glycolysis is a crucial pathway for rapid ATP generation under hypoxic conditions, such as the ischemic microenvironment of injured spinal cords [22]. Glycolysis produces ATP and lactate, both of which are essential for neuronal survival and axonal regeneration [48, 49]. Therefore, managing glycolytic metabolism within the injury site is pivotal for recovery from SCI. Our results revealed that glycolytic activity was significantly reduced and that apoptosis was significantly increased in H₂O₂-damaged neuronal cells and a rat model of SCI. This observation is consistent with previous studies demonstrating that metabolic suppression exacerbates secondary injury [50]. Notably, ADSCs reversed this metabolic decline. This work, from a metabolic perspective, investigated the neuroprotective effects of ADSCs after SCI.

The therapeutic potential of ADSCs in SCI has been attributed to their neurotrophic and immunomodulatory properties [51–53]. In this study, co-culture with ADSCs upregulated the expression of key glycolytic enzymes and increased lactate production in damaged neurons. These beneficial effects were attenuated by the administration of 2-DG, a glycolysis inhibitor, confirming that enhanced glycolysis is indispensable for ADSCs-mediated neuroprotection. This finding resonates with emerging evidence that MSCs regulate cellular bioenergetics to alleviate renal ischemia/reperfusion injury [13]. RNA sequencing revealed that traumatic brain injury (TBI) disrupted many genes involved in metabolic pathways. MSCs treatment was found to correct energy homeostasis and ATP production after brain injury by normalizing 81% of the genes associated with glycolysis and gluconeogenesis [54]. Recent studies have shown that MSCs exert their immunoregulatory and therapeutic effects through triggering metabolic alterations in immune cells [55, 56]. However, the mechanism by which MSCs regulate glycolytic metabolism to exert neuroprotective effects in SCI has not yet been reported.

Our previously published work indicated that ADSCs reduce lesion size and promote functional recovery after SCI mainly via TGF-β1 secretion [57], and consistent with literature reporting TGF-β1 as a potent inducer of FOXK1 expression/activation in multiple cell types (e.g., gastric cancer cells, hepatocytes, and renal tubular epithelial cells) [27, 58, 59]. Our data directly demonstrate that blocking TGF-β1 signaling abolishes ADSC-induced activation of the FOXK1-HK2 axis and subsequent glycolytic enhancement, confirming TGF-β1 as a critical paracrine mediator.

HK2, the rate-limiting enzyme in glycolysis, plays a pivotal role in governing glycolytic flux and mitochondrial function, thereby acting as a crucial regulator of metabolic adaptation [60, 61]. TBI increased serum levels of ubiquitin C-terminal hydrolase-L1 (UCH-L1) and glial fibrillary acidic protein (GFAP) while decreasing hexokinase (HK) expression compared with non-injured controls [62]. The neuroprotective potential of resveratrol is mediated by SIRT1-dependent enhancement of neuronal glycolysis in the brain, which is concomitant with increased expression of the glucose transporter (GLUT1) and HK2 [63]. In our study, HK2 emerged as a key downstream effector of ADSCs. ADSCs treatment led to a significant upregulation of HK2 expression at both the mRNA and protein levels, and this upregulation was closely associated with improved neuronal viability and reduced apoptosis. Further mechanistic investigations revealed that the transcription factor FOXK1, a known regulator of glycolytic metabolism [64, 65], directly controls HK2 expression. Dual-luciferase reporter assays and Cut&Run assay confirmed that FOXK1 enhanced HK2 transcription, whereas siRNA-mediated FOXK1 knockdown abolished ADSCs-induced HK2 upregulation. This knockdown also led to the suppression of glycolytic activation, as evidenced by reduced lactate production, and impaired neuroprotection, as indicated by decreased expression of MAP2/GAP43, which are markers of neuronal integrity and regeneration. In vivo, silencing FOXK1 via AAV-shFOXK1 reversed the therapeutic benefits of ADSCs, including impaired locomotor recovery, increased tissue cavitation, reduced neuronal survival, and disrupted myelin regeneration. These in vivo findings, in conjunction with our in vitro results, firmly establish the FOXK1-HK2 axis as a pivotal mediator of the metabolic reprogramming effects of ADSCs in the context of SCI. These findings are consistent with previous reports that FOXK1 modulates glycolysis by regulating HK2 and lactate dehydrogenase in adipocytes [29]. However, our study extends this regulatory axis to a completely novel context, namely neuronal repair in SCI, highlighting its broader significance in tissue repair and regeneration. This discovery serves as a crucial bridge, connecting stem cell therapy with the transcriptional regulation of energy metabolism.

## Conclusions

In summary, our findings demonstrate that ADSCs are capable of restoring neuronal glycolysis through the FOXK1-HK2 signaling pathway, thereby replenishing the energy supply and facilitating tissue regeneration. This research not only uncovers a previously unrecognized metabolic mechanism accounting for the neuroprotective effects of ADSCs but also identifies the FOXK1-HK2 pathway as a highly promising therapeutic target. Moreover, this study provides a scientific basis for integrating stem cell therapy with metabolic modulation strategies to improve the therapeutic outcomes of SCI treatment. Strategies to activate FOXK1 or overexpress HK2 might synergize with ADSCs transplantation to amplify glycolytic flux, particularly in the hypoxic SCI microenvironment where oxidative phosphorylation is compromised. Such combinatorial therapeutic approaches have the potential to significantly improve the efficacy of current stem cell-based therapies for SCI, offering new hope for patients suffering from this debilitating condition.

## Supplementary Information

Below is the link to the electronic supplementary material.

Supplementary Material 1

Supplementary Material 2

Supplementary Material 3

## Data Availability

The data that support the findings of this study are available from the corresponding author upon request.
