# Supplementary material for: Adipose tissue-derived stromal cells enhance glycolytic metabolism in injured nerve cells via the FOXK1-HK2 axis for spinal cord injury repair
Source: J Transl Med. 2026 Mar 17;24:582. doi: 10.1186/s12967-026-07958-w (PMC13107693; doi:10.1186/s12967-026-07958-w)
Supplement: Supplementary file 1 — Supplementary Material 1 [file 12967_2026_7958_MOESM1_ESM.doc]

**Supplementary material S2:** Complete List of RNA-seq-Derived Differentially Expressed Genes

| **Cross** |
| --- |

| Trip10 | LOC102550890 | LOC100911515 |
| --- | --- | --- |
| NONMMUG006859 | Plod2 | Eno2 |
| Cox4i2 | Hspa4l | Cxadr |
| Scd2 | Bnip3 | Tmem51 |
| RGD1563601 | Slc2a1 | Hunk |
| Lamb3 | Gpi | Acp5 |
| LOC100362783; Tsc22d4 | Slc16a3 | LOC100911625 |
| Ero1a | LOC100910973; RGD1566254 | Tpi1 |
| Naa50 | Abca1 | Slc38a4 |
| Fzd5 | Plod1 | Loxl2 |
| LOC681458; LOC689095 | Hk2 | Slc4a8 |
| Scgb2a2 |  |  |

**H_2_O_2_ vs. normal**

| Slc2a3; LOC100909595 | Cdon | Srgap3 |
| --- | --- | --- |
| Lrp5 | Camk4 | Map3k8 |
| Spp2 | Sgk1 | Clstn1 |
| LOC102550455 | Agfg2 | Srebf2 |
| Slc2a3 | Tmem102 | Hoxc4 |
| Slit3 | Atp2b4 | Lpin1 |
| Vegfa | Ppfibp2 | Myt1 |
| Ccdc187 | Gstm1 | Mllt6 |
| Tigit | Pgf | Olr158; LOC100910597 |
| LOC100911906 | Cep44 | Ip6k1 |
| Rab11fip5 | Tmtc1 | Cacna1s |
| Tbx20 | Tef; LOC100910463 | NONMMUG000169 |
| Hsd11b1 | Galns | Slc6a2 |
| Spata17 | Adm | Adora2a |
| CYTB | Boc | S100a4 |
| Tpd52l1 | Chrna3 | Tbc1d23 |
| Klhl29 | Rgs4 | Racgap1 |
| Fam117a | Crebl2 | Slc16a10 |
| COX2 | Patz1 | LOC100912399 |
| Kdm3a | LOC102547216 | Llgl1 |
| Ubiad1 | Tmem252 | Ppp1r26 |
| ND2 | RGD1307100; RGD1309985; RGD1561393; ENSRNOG00000026660; ENSRNOG00000032689 | Spr |
| Shmt2 | Socs2 | PHOX2B; Phox2b |
| Casz1 | Galk1 | Il17c |
| Micalcl | Cdk19 | Hemgn |
| Gucy1a3 | Pdzd2 | Rbpms |
| ND1 | Olr556 | ND4; ND4L |
| Kif18b | Pc | Igsf9 |
| Tmem164 | Tesc | Fads2 |
| Fam78a | Lilra5 | ND5 |
| Olr542 | Gjd2 | Psmd10 |
| Zfpm1 | LOC679873 | Kifc2 |
| ND6 | Slc18a1 | Slc29a1 |
| Scd1 | Ano6 | LOC102546929 |
| Cd6 | LOC100362142 | Pacs2 |
| Ube2o | Fads3 | Sema4d |
| LOC685699 | Slco3a1 | Ikbkap |
| Ccdc73 | Rab11fip3 | Sprr1a |
| Rgs5 | S100a5 | Hand1 |
| Ddc | Ldlrap1 | Gjb5 |
| Lrp4 | Olr475 | Ccdc84 |
| Sh2d3c | Parva | Fxyd2 |
| Cyb5d1 | RGD1560795 | Slc39a10 |
| Lnpep | Gcgr | Kif26b |
| Cerk | ND3 | Celf3 |
| Dhcr7 | Lcat | Sc5d |
| Srebf1 | Syt9 | LOC100362109 |
| Ppargc1b | L3mbtl4 |  |

**ADSCs vs. H_2_O_2_**

| Med10 | Arhgef40 | LOC100364062 |
| --- | --- | --- |
| Nppb | LOC619574 | Hivep2 |
| Vstm1 | Cdc34 | Gapdh-ps2 |
| Nfat5 | Papd5 | Fosl2 |
| Mreg | RGD1309621 | Stc2 |
| Ostf1 | Adarb1 | Frmd4a |
| P4ha2 | RGD1565560 | Ccnyl1 |
| Limk2 | Ercc1 | Spopl |
| Polr2e | LOC102552640; Rcor2 | Slc17a4 |
| Zdhhc3 | P4ha1 | Klf4 |
| Cyp2s1 | Ntmt1 | Abl2 |
| Kdm6b | Mt1a | Cacna2d1 |
| Nfatc4 | Olr324 | Micall2 |
| Map4k4 | Hmox1 | Slc41a2 |
| Pkm | LOC102554695; LOC100909544 | Serpinb2 |
| Nptxr | Mir3584 | Plod3; Mir702 |
| Maff | Arhgap17 | Dip2b |
| Bhlhe40 | Rnf149 | Faap100 |
| Fosb | Pygl | Tle3 |
| Zyx | Ptrh1 | Qk |
| Bdh1 | Msi2 | RT1-CE4 |
| Entpd2 | Smox | Ier5 |
| Myd88 | Fstl3 | Fam168b |
| Evi5l; ralo | Tpm4 | Elk3 |
| Olr1652 | Cdc42se1 | Hspb8 |
| Trim25 | Nucb2 | Zswim4 |
| LOC100912218; Itprip | Mst1r | Casp3 |
| Lpcat3 | Hnrnpul2 | Alkbh5 |
| LOC100912537 | Gng12 | Chsy3 |
| Zfp566 | Brd2 | Sapcd2 |
| Egln1 | Sec16b | Prl6a1 |
| Apitd1; LOC100360180 | Tmem170a | Dusp10 |
| Comt | Tirap | Sppl3 |
| Sh3kbp1 | LOC498316 | Ndel1 |
| 8-Mar | Stk10 | Ddit4 |
| Lcn2 | Ehbp1 | LOC100912070 |
| RGD1309104 | Thbs4 | Itga3 |
| Rnf126 | Birc7 | Tmcc3 |
| Pde10a | Sv2c | Otud3 |
| Cited2 | Sbsn | LOC100363436 |
| LOC500959 | Fam122a | Rem2 |
| Rab24 | Pdk1 | Rgag4 |
| Gadd45b | Nfil3 | Nt5c |
| Ppa2 | LOC102549726; RGD1566118 | Higd1a |
| LOC100365259; Spty2d1 | Arl16 | Pom121 |
| Mrto4 | Helb | LOC102553405 |
| Mafk | Gsn | Tnfaip3 |
| Cd28 | Myc | Skil |
| Alcam | Helz | Tnfaip8 |
| Pip5k1a | Ptgs1 | Smndc1 |
| Crem | Inppl1 | LOC100362344 |
| Abcf3 | Fxyd5 | Ccnj |
| Slc7a15 | Fbxo42 | Brap |
| Slc23a2 | Serpine1 | Clic1 |
| Odc1 | Btg2 | Stat3 |
| Wdr82 | Fam129a | Cldn4 |
| Eif1 | Esr2 | Olr1766 |
| Yrdc | Baz1a | B4galt3 |
| Wdr91 | Rab3ip | Cd300le |
| LOC102550650 | Rnf19b | LOC102550651 |
| Fbxo46 | Tmod3 | Il18bp |
| Cdhr2 | Noc2l | Kdm5c; LOC102555632 |
| Phlda1 | Zxdc | Uhrf1bp1l |
| Sdk2 | Scd4 | Ykt6 |
| Snap23 | Htra3 | Slc12a9 |
| Emilin1 | Arl5b | Fbxo32 |
| Mex3b | Ankrd13a | Zfp57 |
| LOC102554096; ENSRNOG00000042583 | Stfa2l2 | Sgpp1 |
| Bmp3 | Sncb | Dusp5 |
| Sphk1 | Bsn | Lysmd3 |
| LOC100363171 | RGD1306091 | Prss22 |
| Olr1130 | Tenm2 | LOC688459; LOC688548 |
| Tmub1 | Neu2 | Abcc6 |
| Doc2b | Zfand5 | Pgk1 |
| Nrep | Tnfrsf26 | Zfp52 |
| Tbc1d14 | Mxd1 | Slc5a2 |
| RT1-CE15 | Ipo5 | Pparg |
| Hcfc2 | Bdkrb2 | Ncam1 |
| RGD1562690 | Mapkapk2 | Taf1a |
| Prkx | Plin2 | Tsc22d2 |
| Celf4 | Anxa8 | Cldn6 |
| Macrod2; LOC102548740 | Safb2; LOC100911466 | Aldoa |
| Pepd | Stc1 | Asxl1; ENSRNOG00000023335 |
| Txndc5 | Bag2 | Qk; LOC100910212 |
| LOC102546505 | Azin1 | Sidt2 |
| Ldha | Ctnnd1 | Bmp1 |
| Dyrk3 | Mapk14 | Csf1 |
| Flna | Klf6 | Csrnp2 |
| Olr1248 | Vopp1 | Cited1 |
| Mtap | Fnbp1l | LOC102554994 |
| LOC102554012 | Gabarap | Dbx1 |
| Il17ra | Ctdp1 | Acly |
| LOC102549694 | Bhlhe41 | Tmsbl1 |
| Sdc4 | Dixdc1 | Pip5k1b |
| Mmp13 | Vom1r110 | Ddi2 |
| Pgam1 | Vtcn1 |  |
