# Supplementary material for: Adipose tissue-derived stromal cells enhance glycolytic metabolism in injured nerve cells via the FOXK1-HK2 axis for spinal cord injury repair
Source: J Transl Med. 2026 Mar 17;24:582. doi: 10.1186/s12967-026-07958-w (PMC13107693; doi:10.1186/s12967-026-07958-w)
Supplement: Supplementary file 2 — Supplementary Material 2 [file 12967_2026_7958_MOESM2_ESM.doc]

**Supplementary material S3:** The raw Western blot images

Fig 3F PC12

| 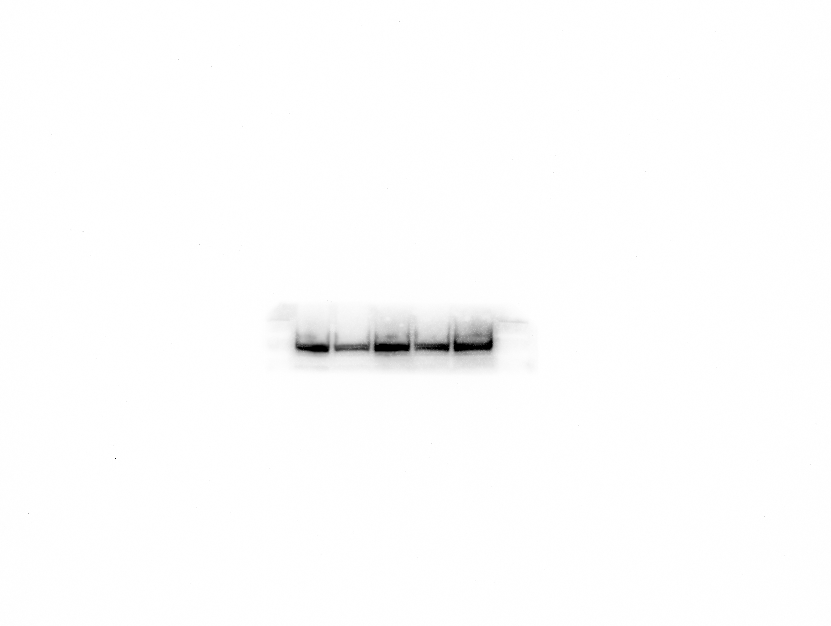 | HK2 102kDa |
| --- | --- |
| 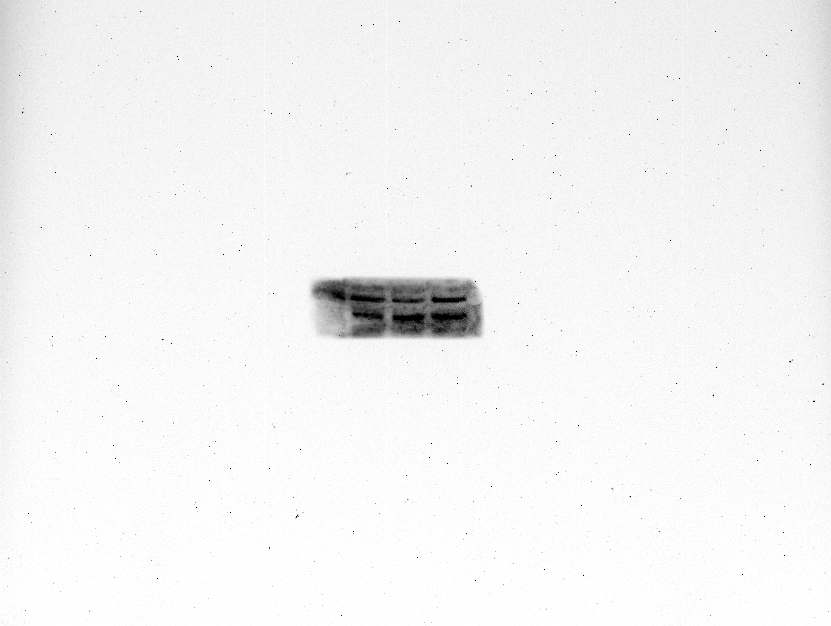 | GPI 55kDa |
| 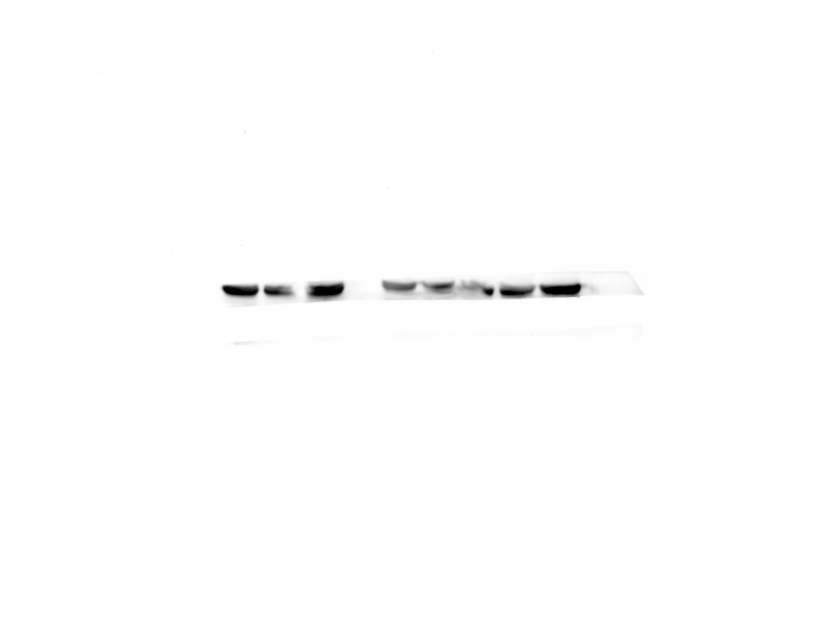 | ENO2 47kDa |
| 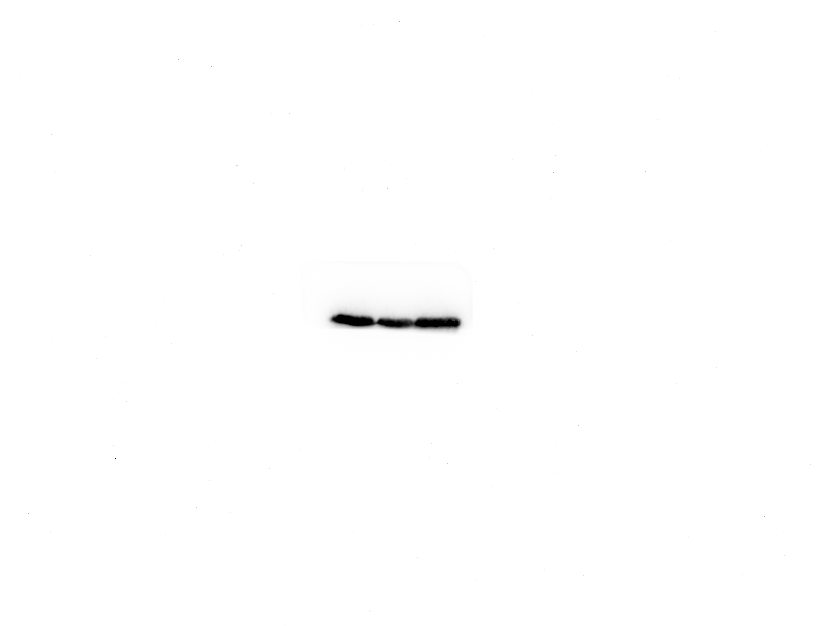 | TPI1 27kDa |
| 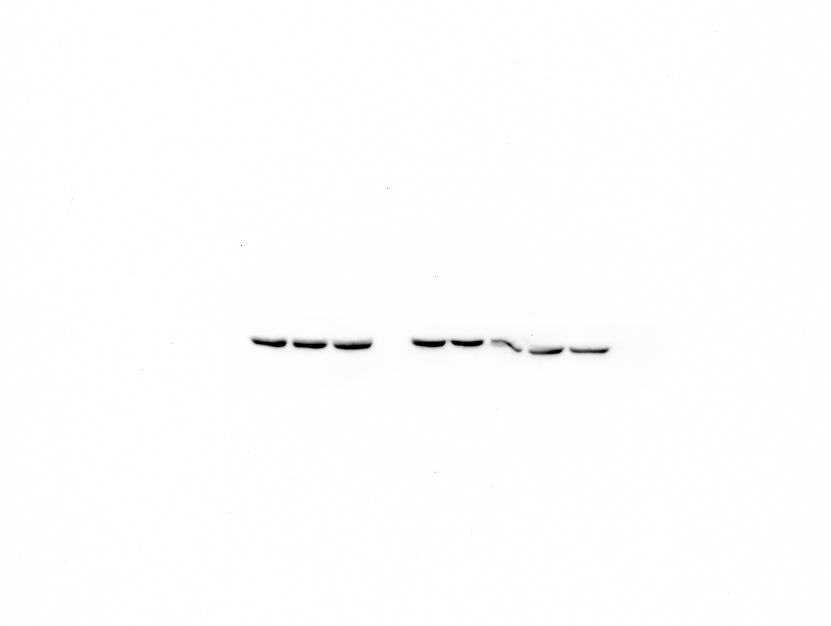 | β-actin 42kDa |

Fig 4E SH-SY5Y

| 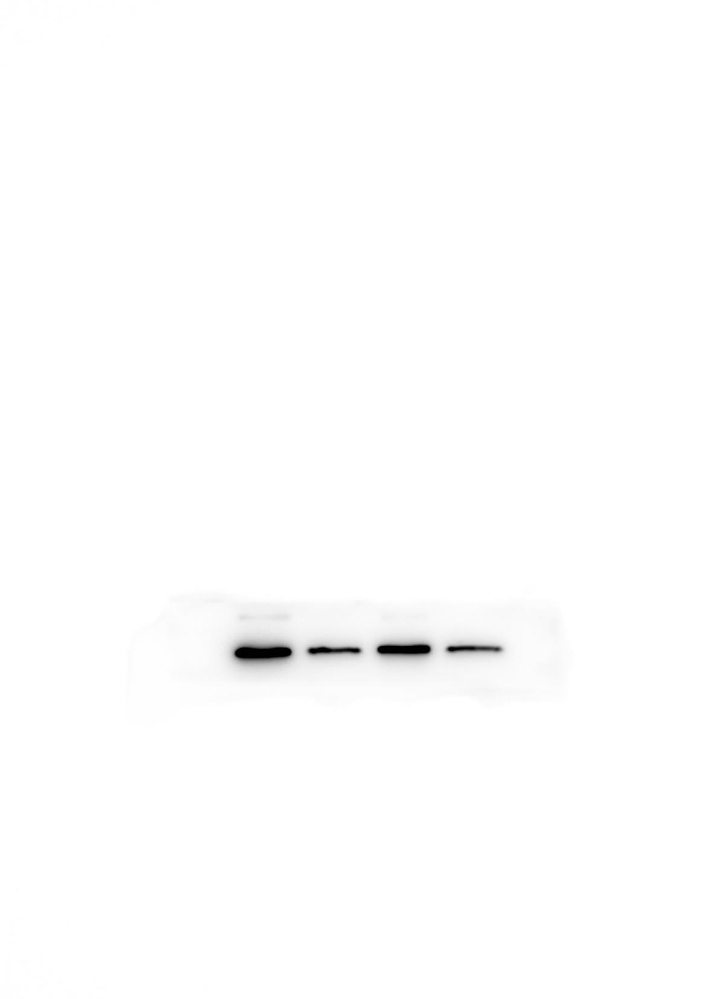 | HK2 102kDa |
| --- | --- |
| 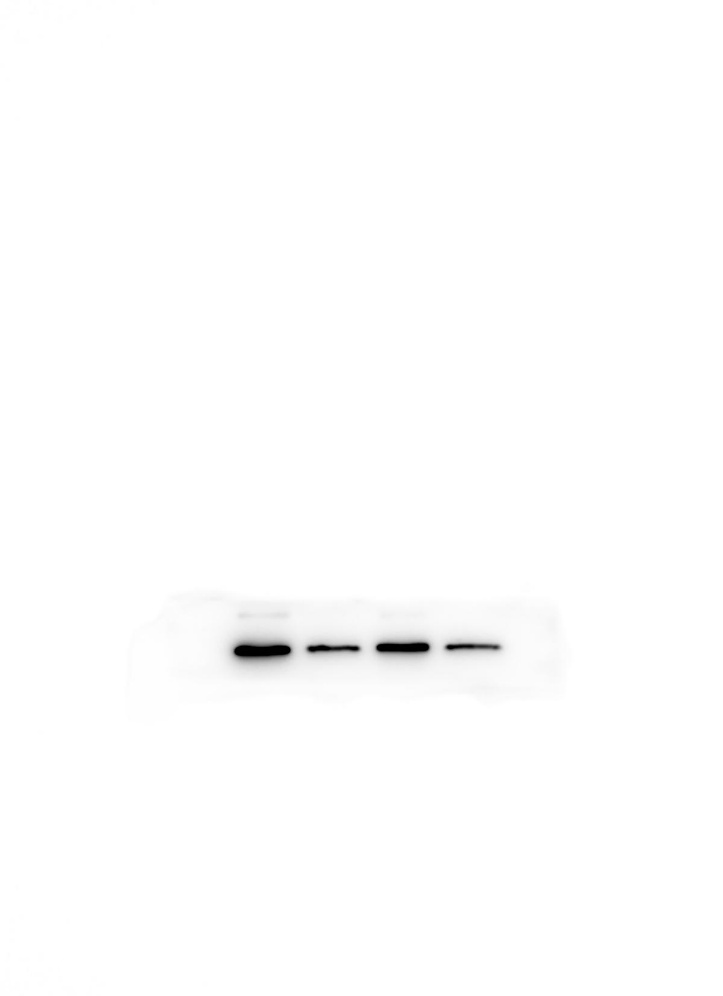 | GAP43 43kDa |
| 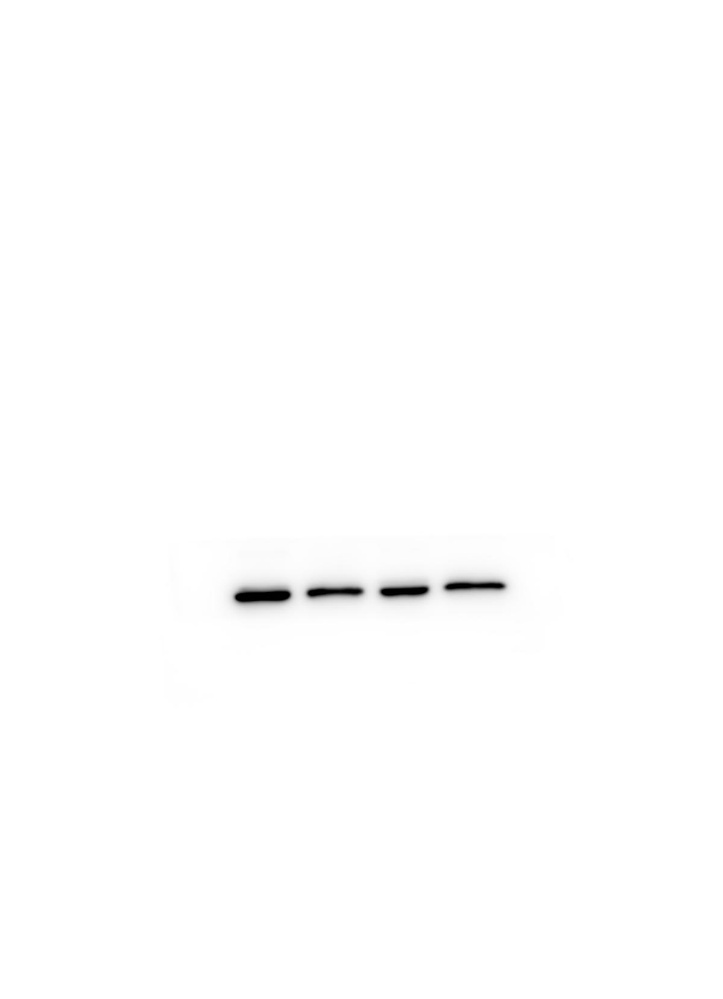 | β-actin 42kDa |

Fig 4L PC12

| 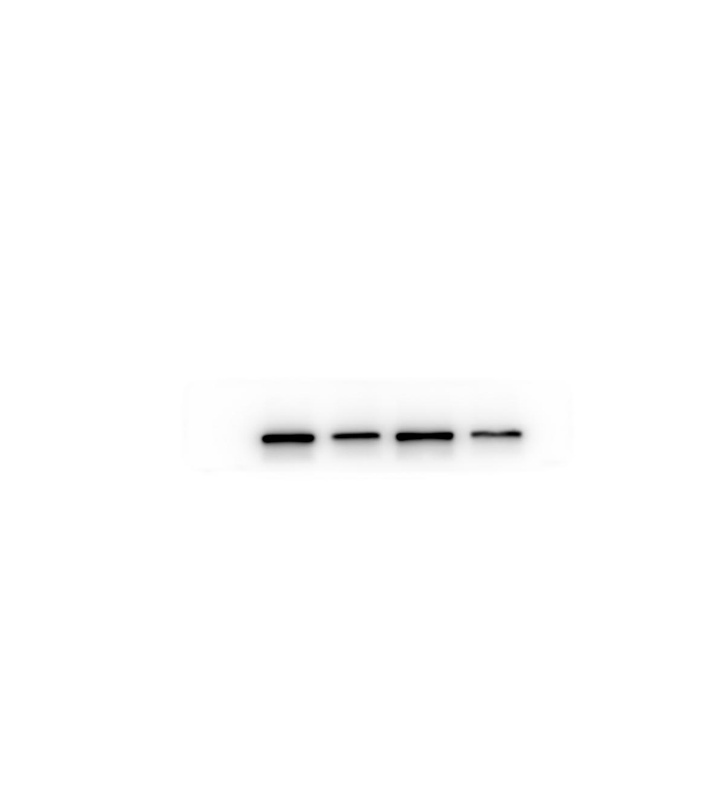 | FOXK1 97kDa |
| --- | --- |
| 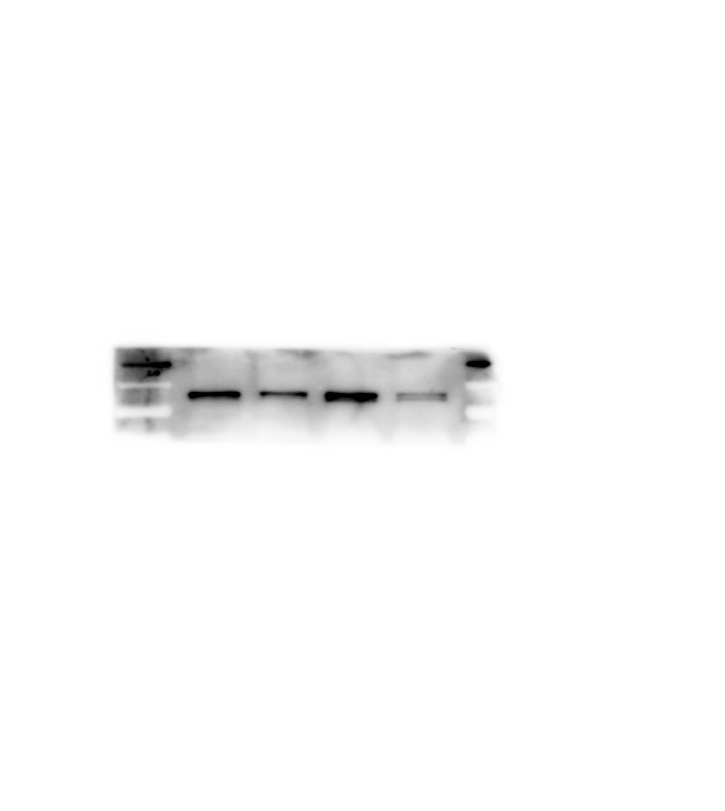 | HK2 102kDa |
| 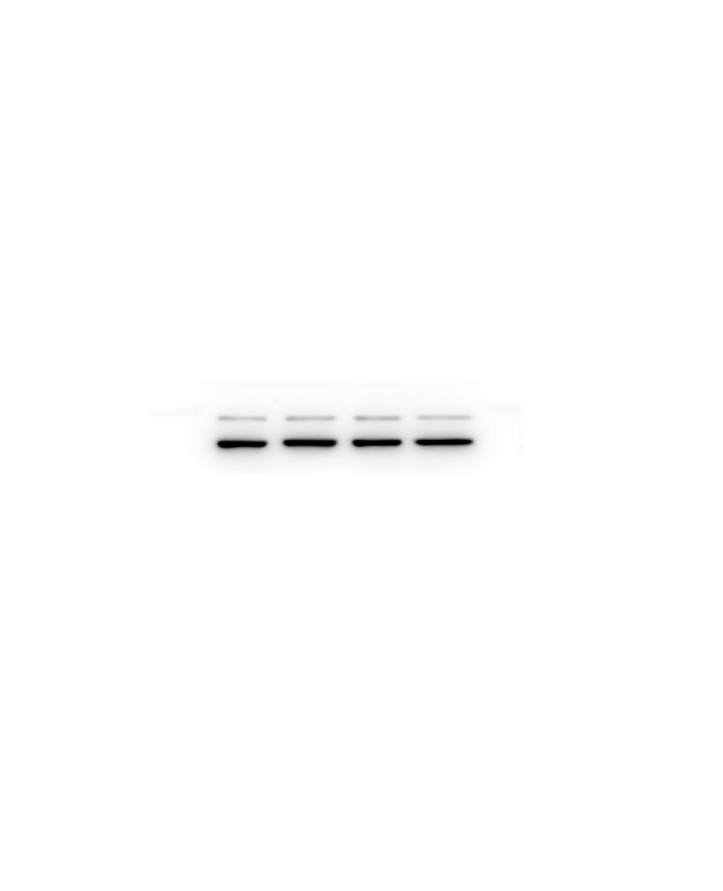 | β-actin 42kDa |

Fig 4L SH-SY5Y

| 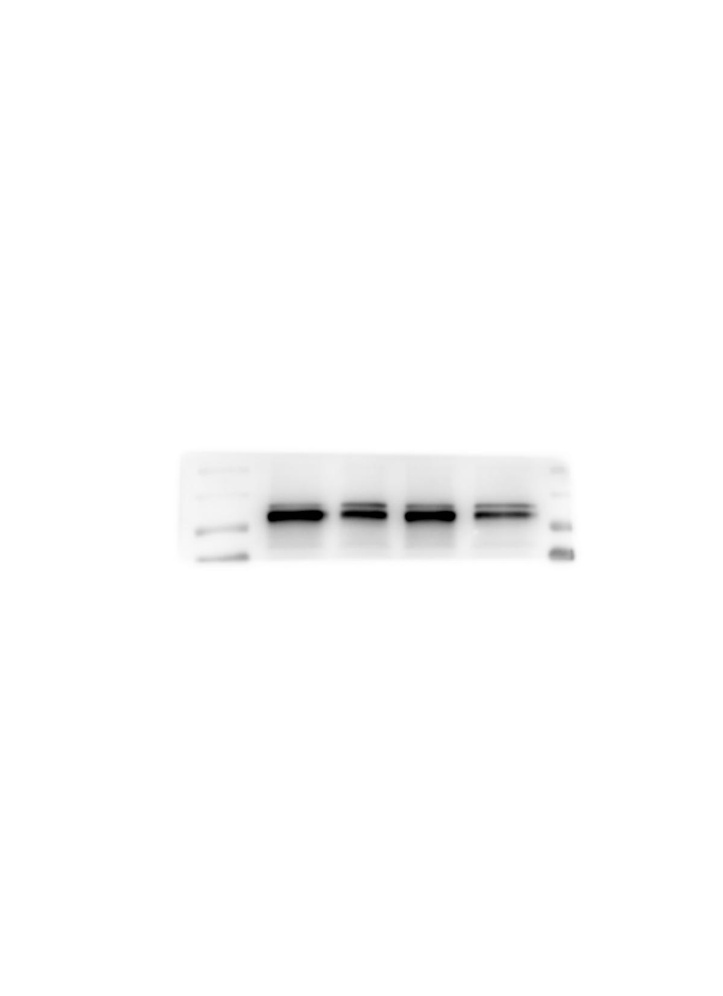 | FOXK1 97kDa |
| --- | --- |
| 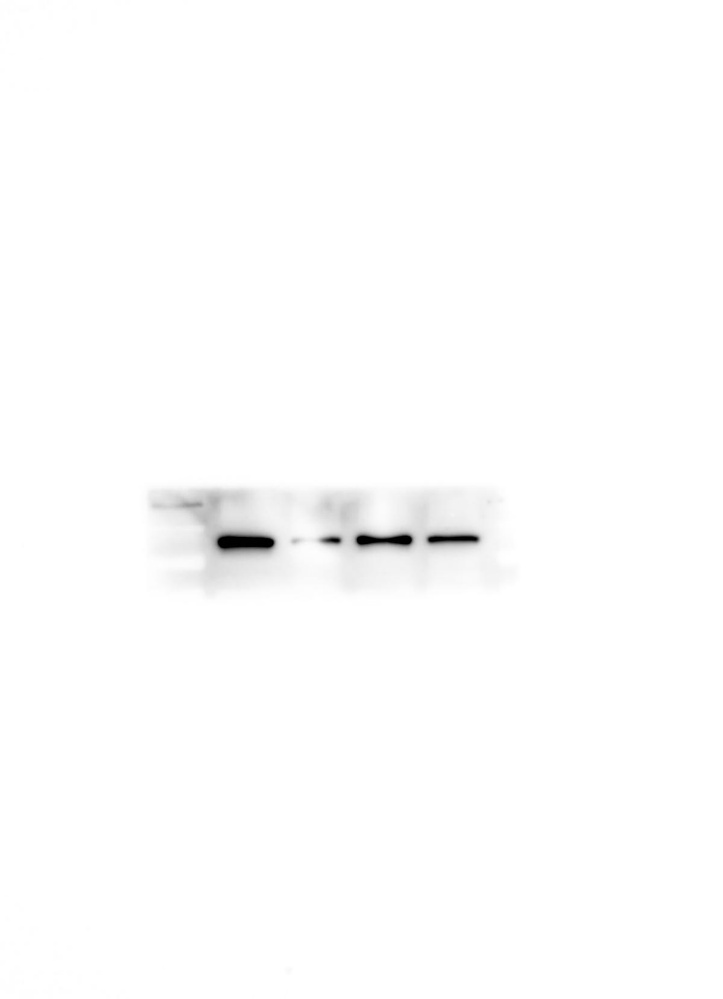 | HK2 102kDa |
| 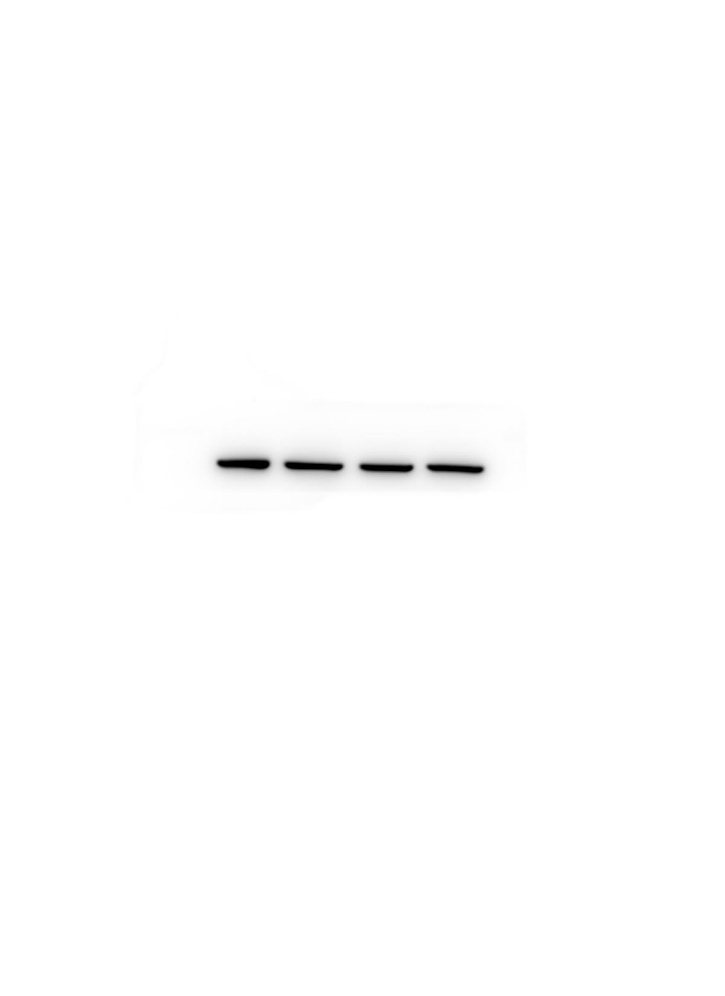 | β-actin 42kDa |

Fig 5F PC12

| 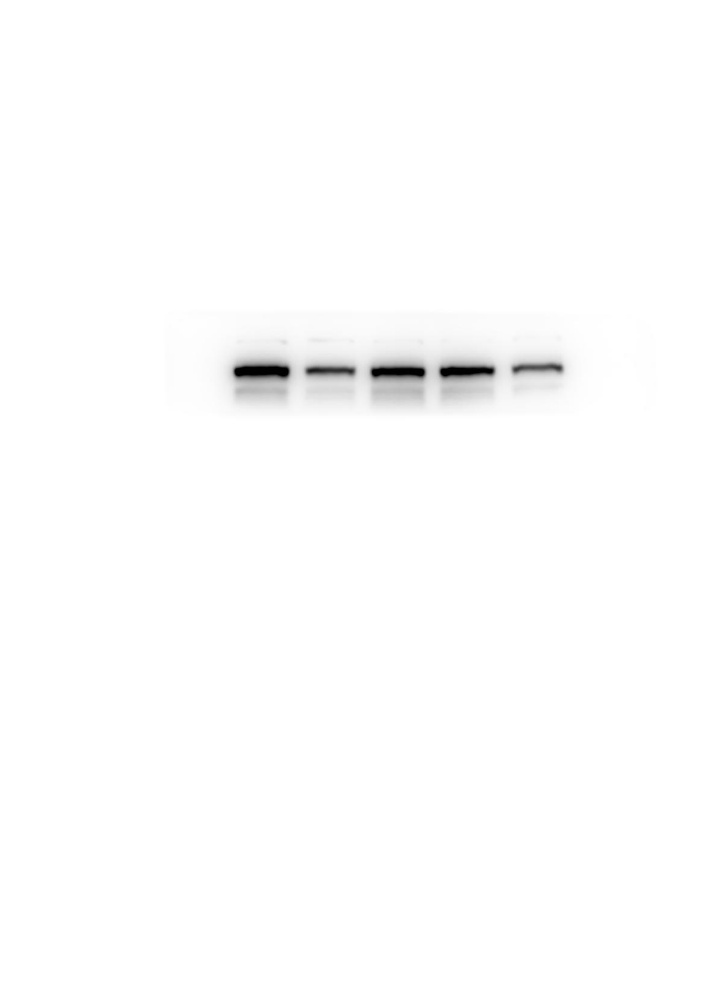 | FOXK1 97kDa |
| --- | --- |
| 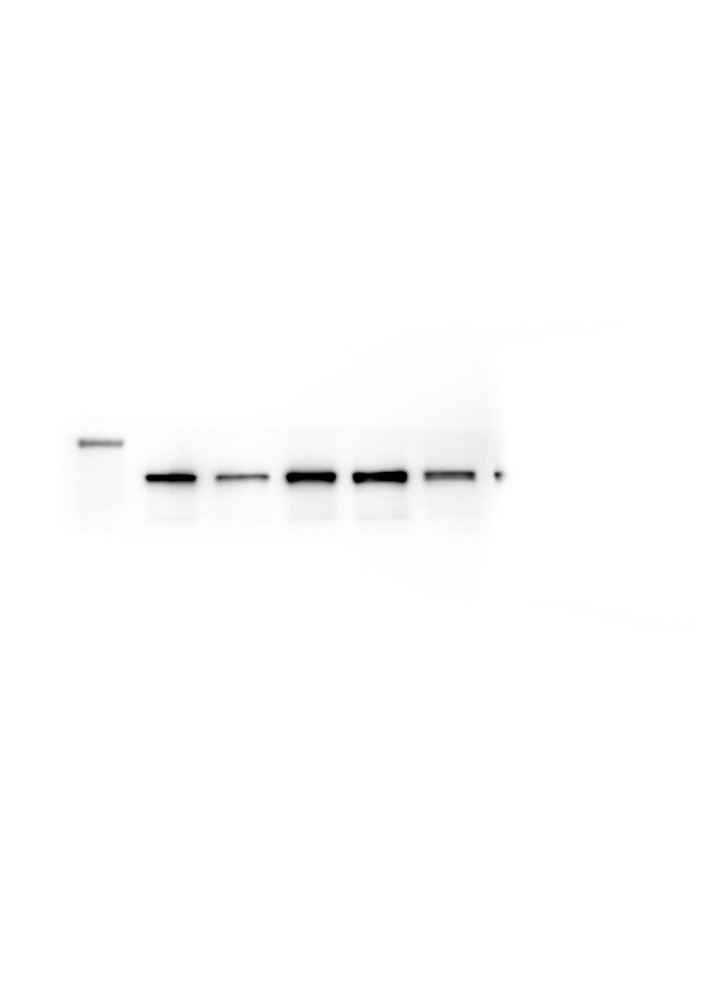 | HK2 102kDa |
| 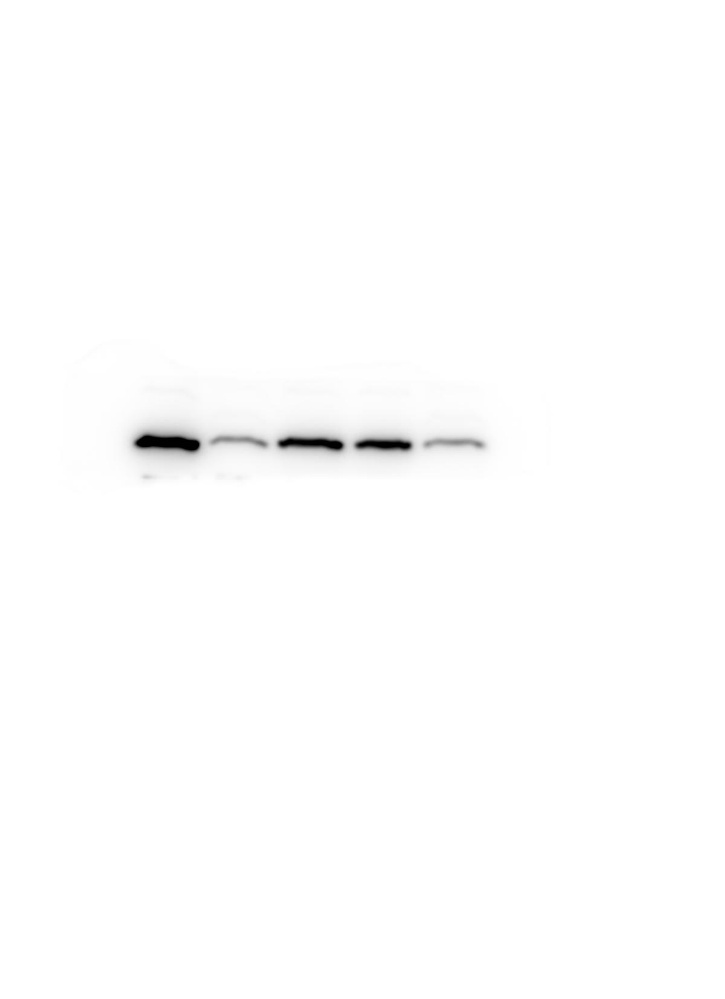 | GAP43 43kDa |
| 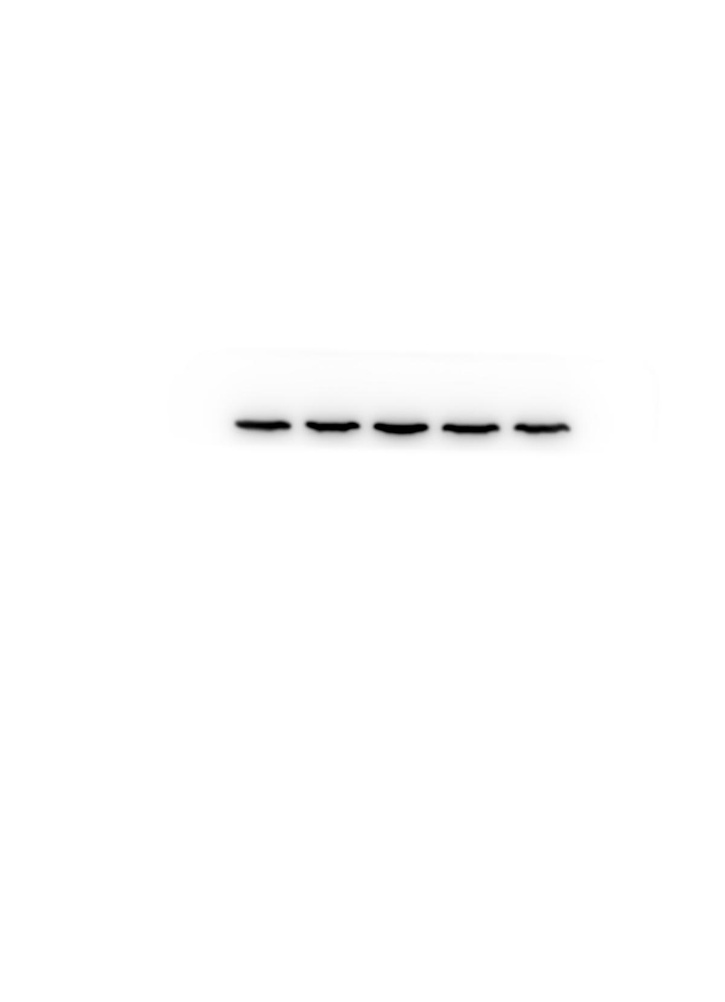 | β-actin 42kDa |

Fig 5F SH-SY5Y

| 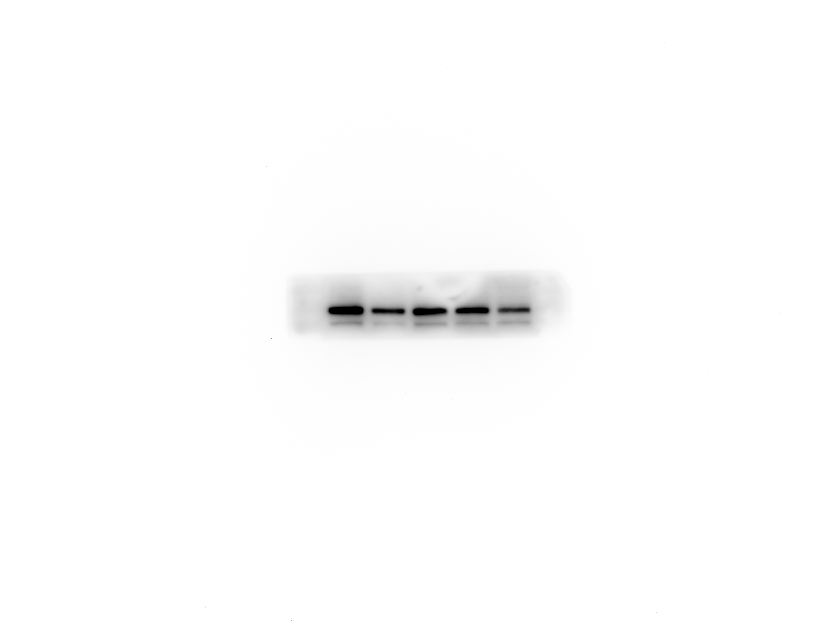 | FOXK1 97kDa |
| --- | --- |
| 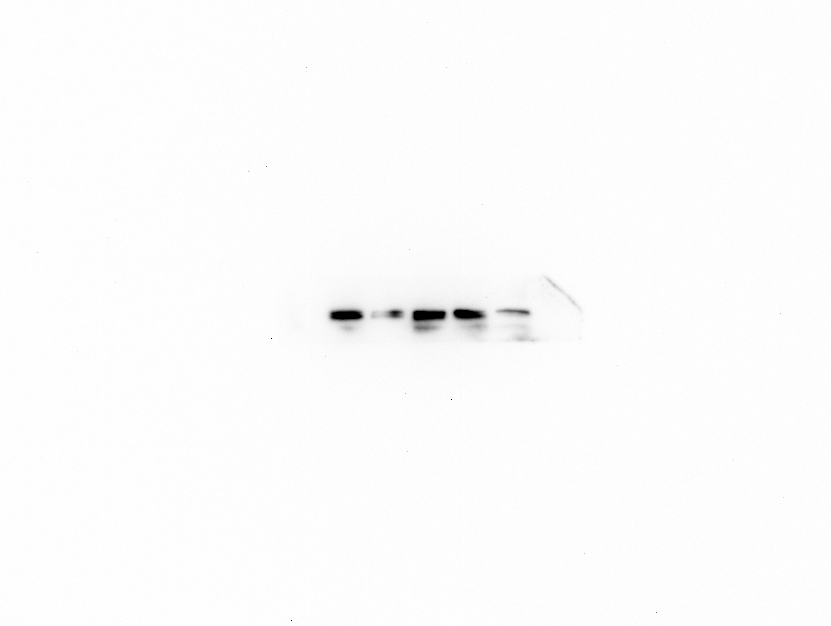 | HK2 102kDa |
| 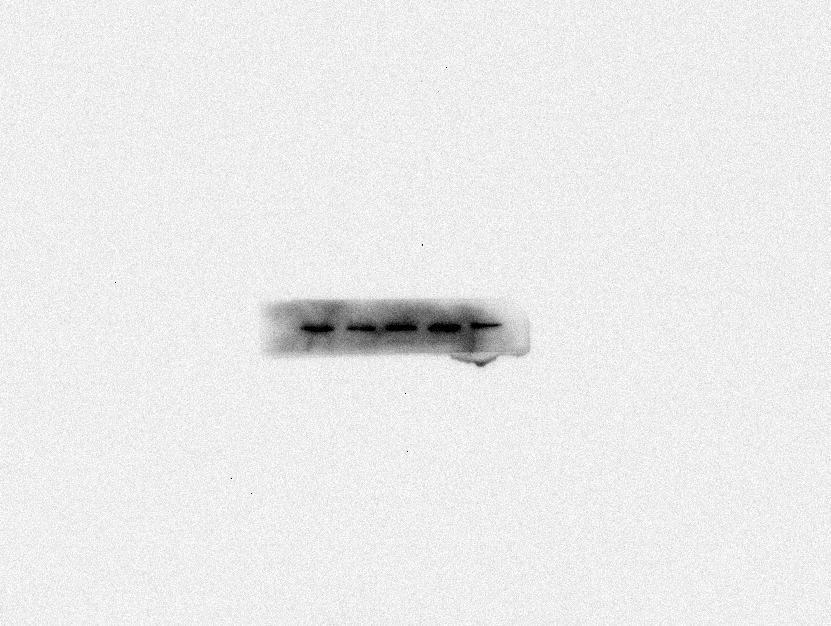 | GAP43 43kDa |
| 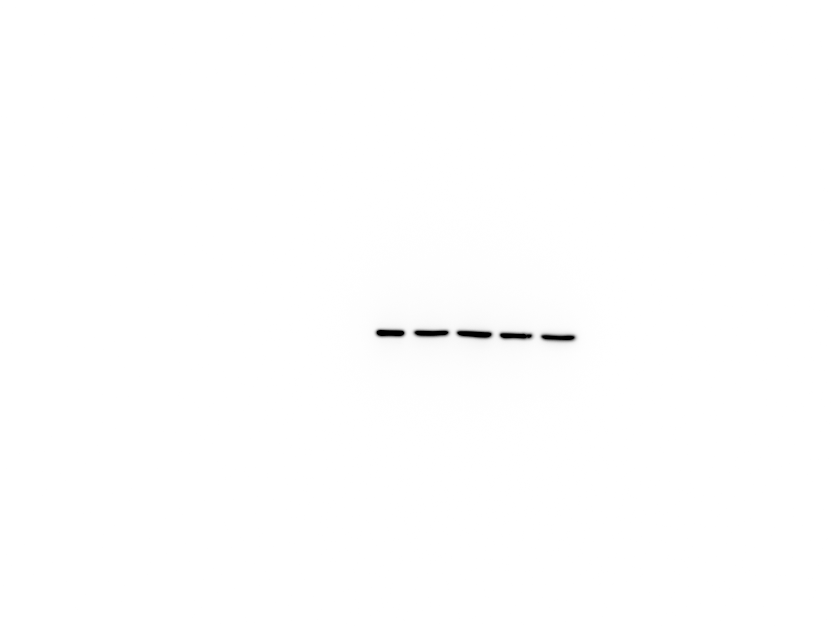 | β-actin 42kDa |

Fig 6B

| 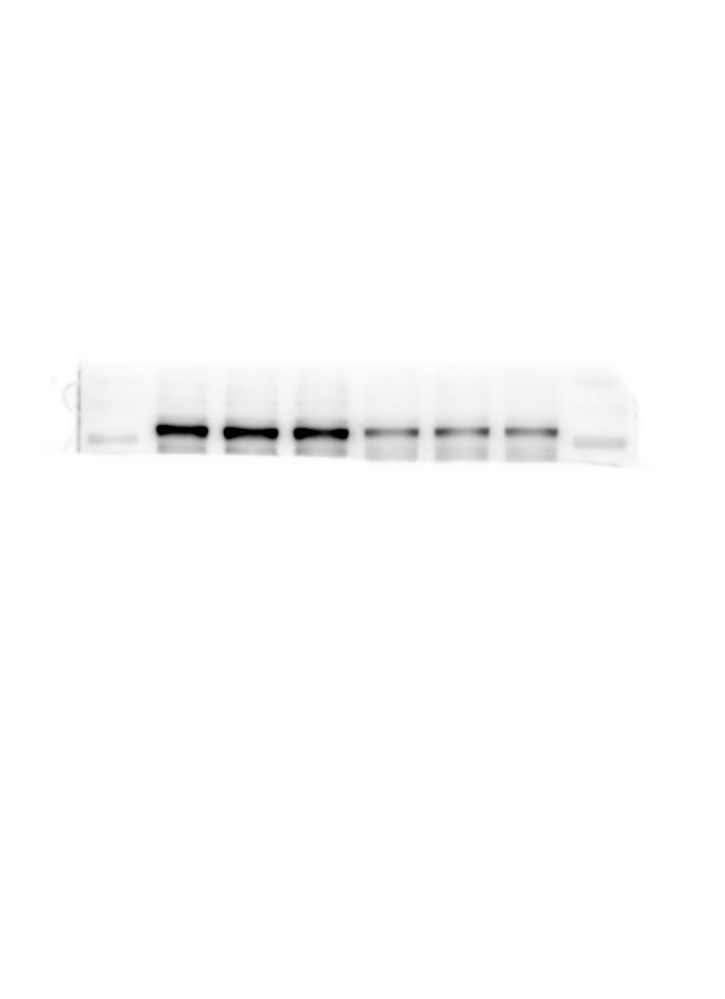 | FOXK1 97kDa |
| --- | --- |
| 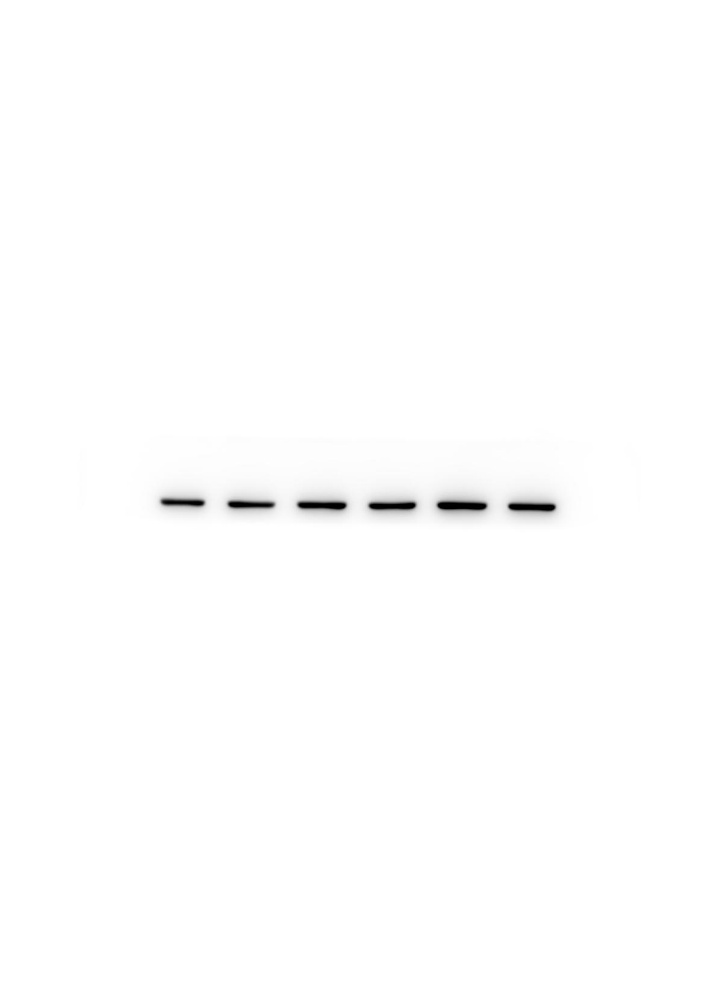 | β-actin 42kDa |

Fig 6E

| 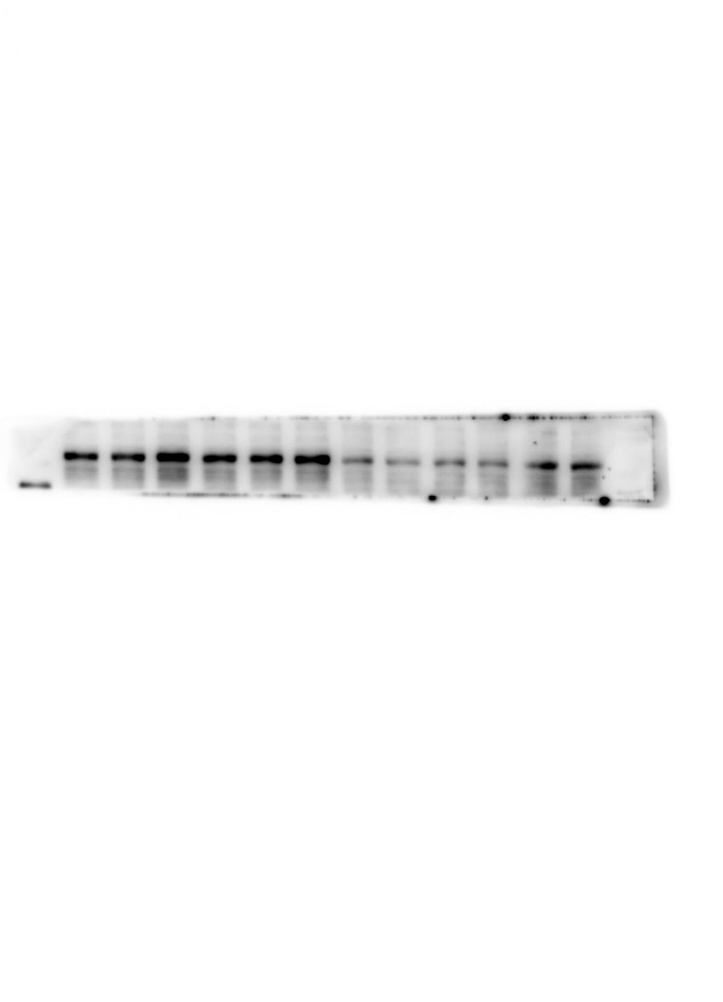 | FOXK1 97kDa |
| --- | --- |
| 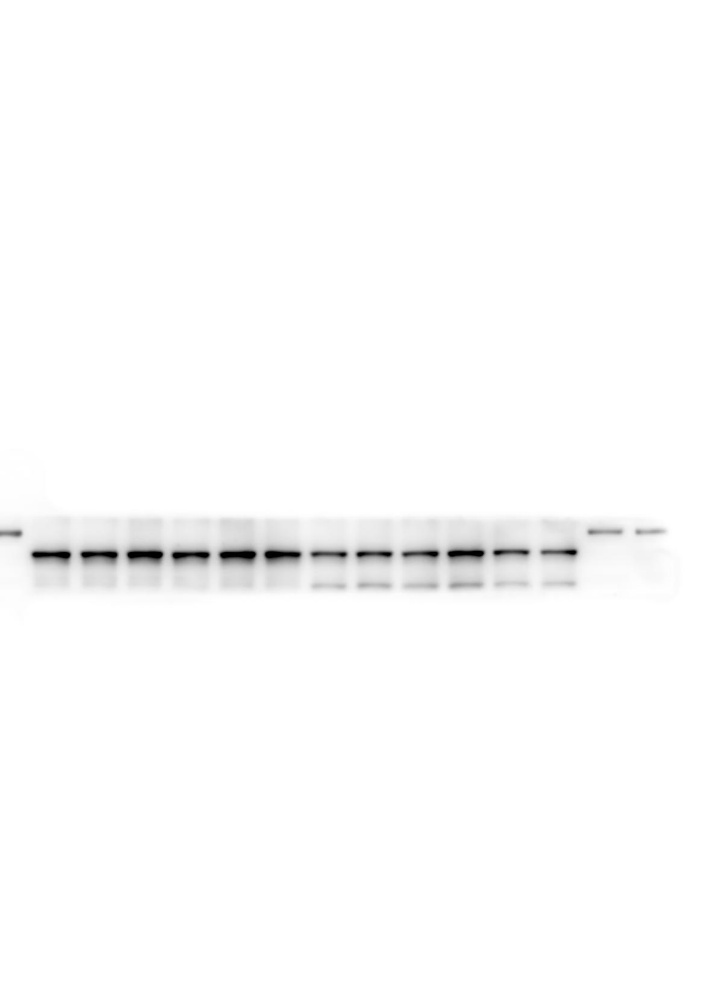 | HK2 102kDa |
| 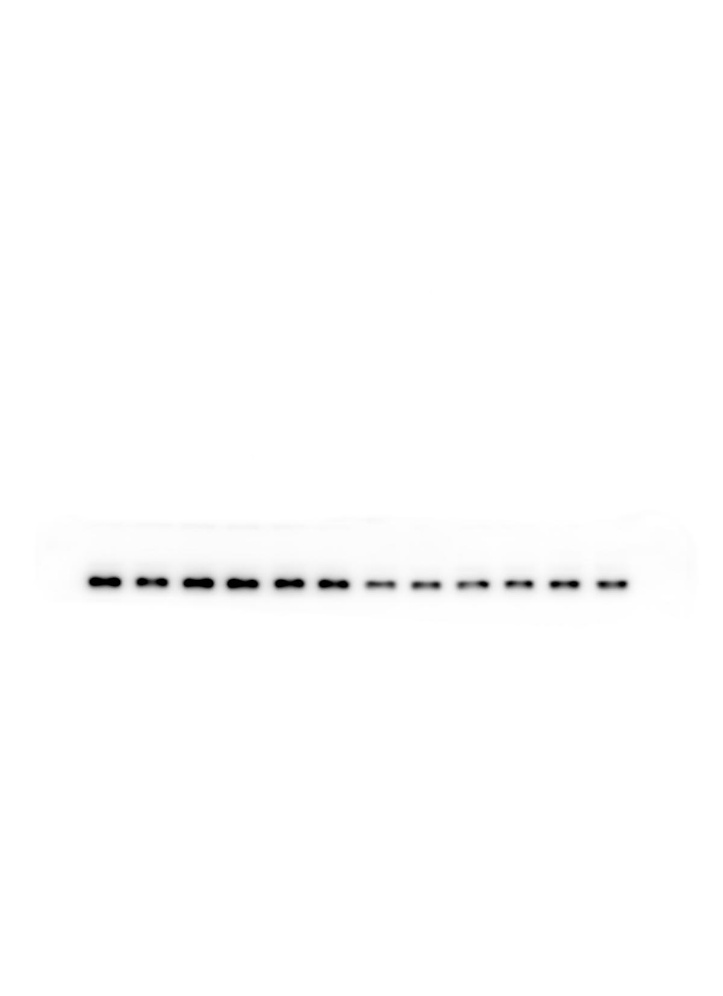 | GAP43 43kDa |
| 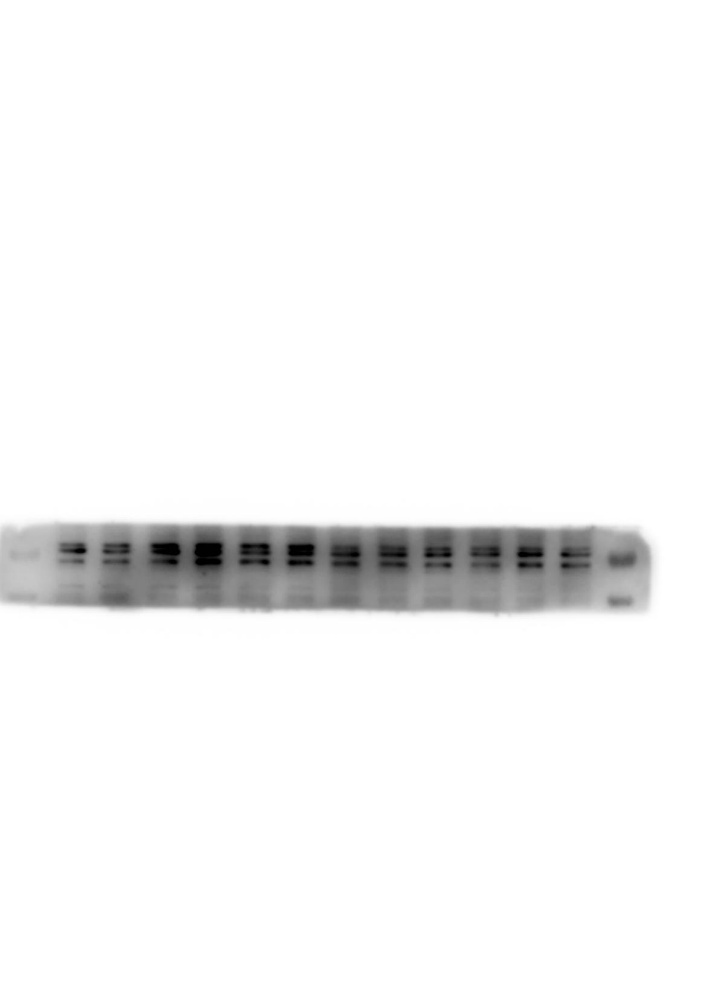 | Bcl-2 26kDa |
| 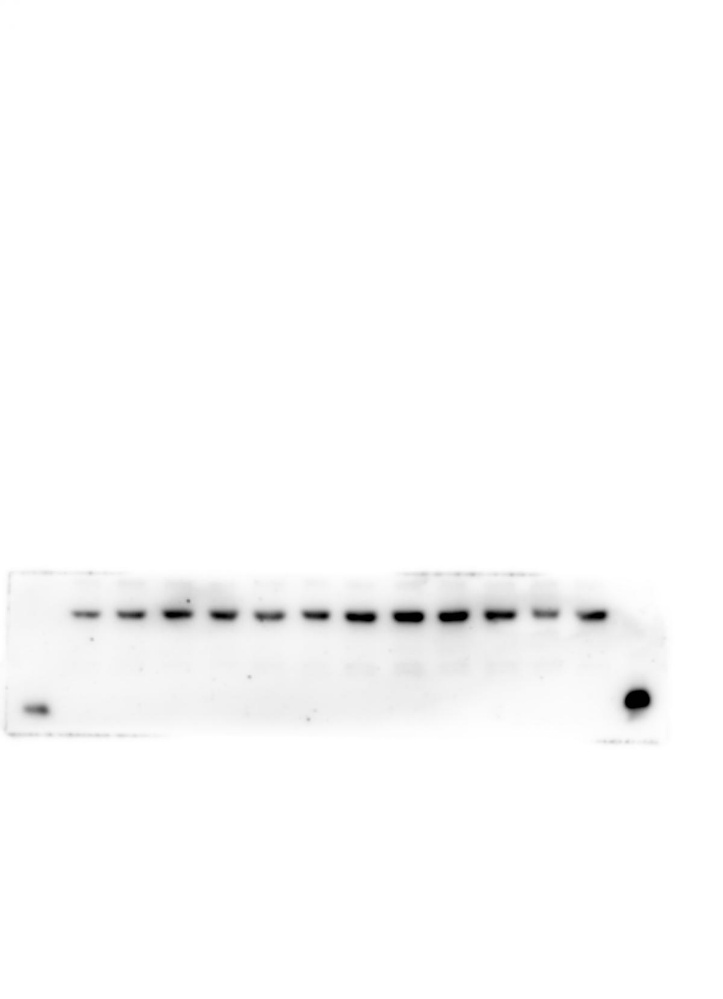 | Bax 21kDa |
| 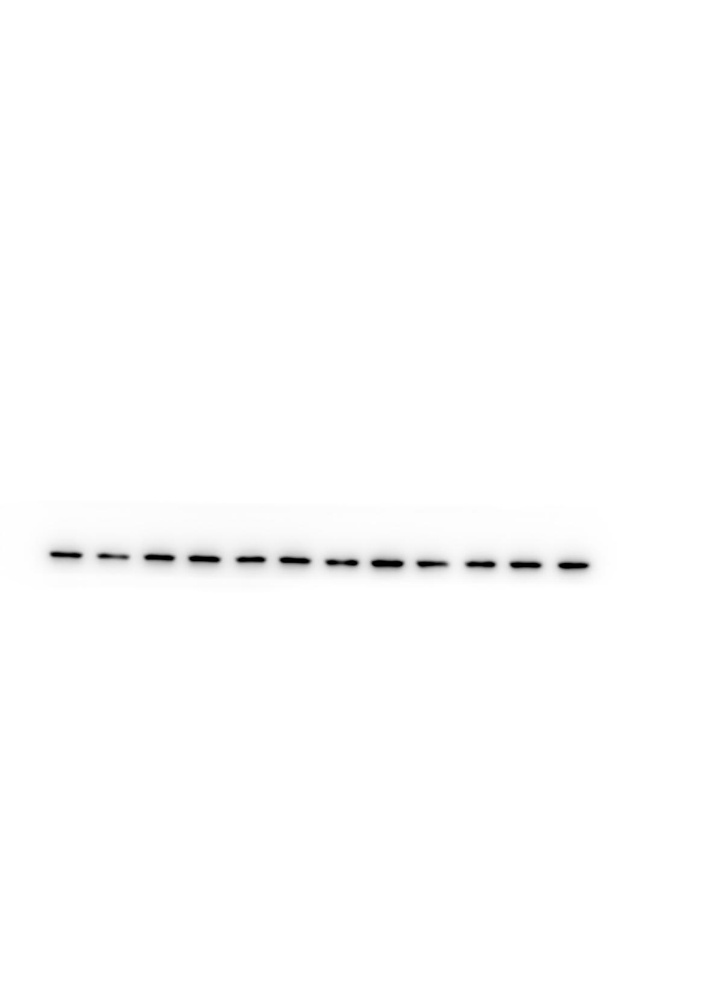 | β-actin 42kDa |

S Fig 3B PC12

| 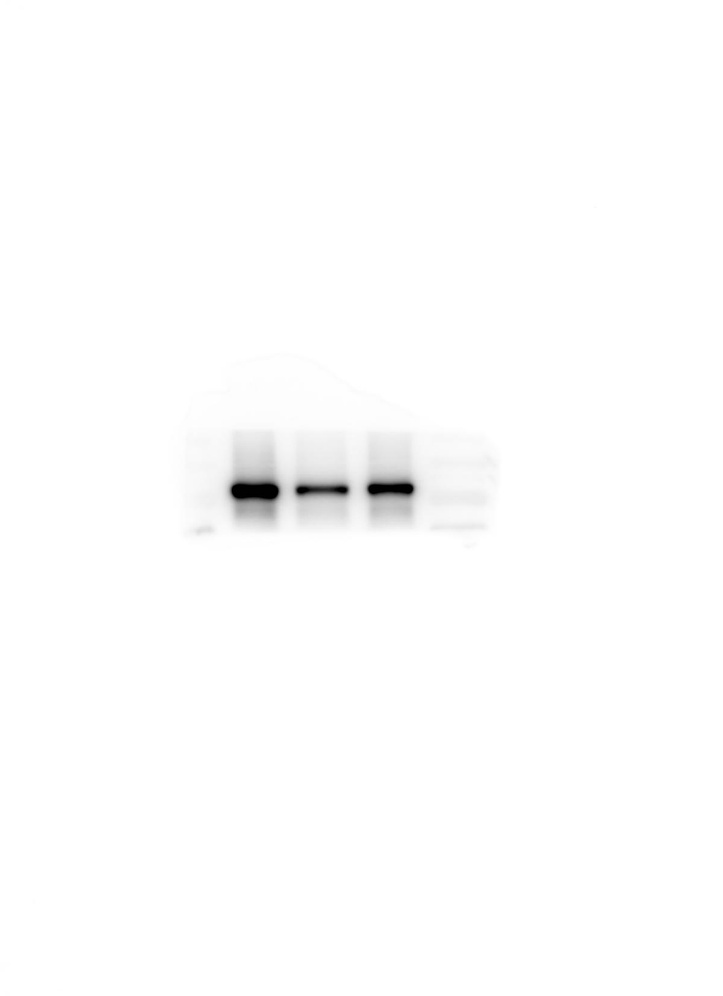 | FOXK1 97kDa |
| --- | --- |
| 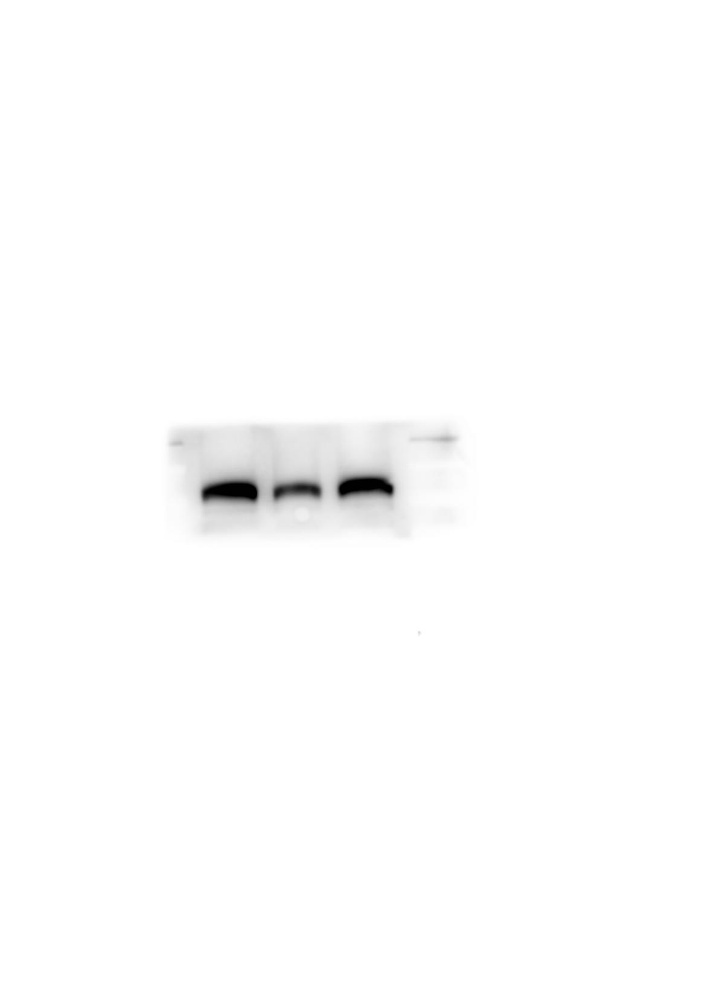 | Hk2 102kDa |
| 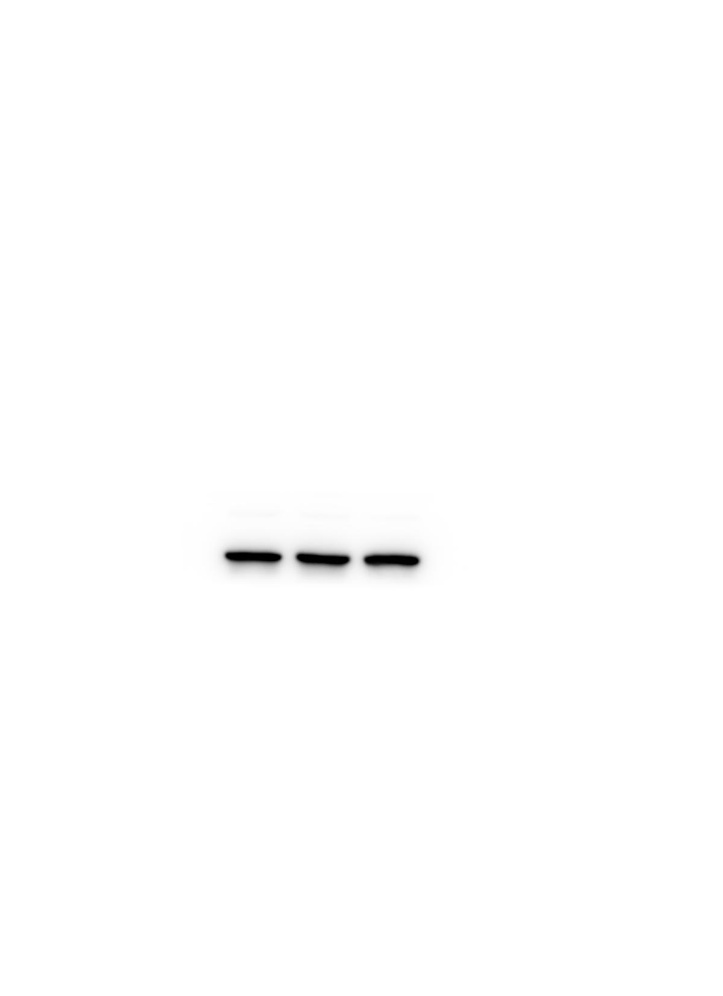 | β-actin 42kDa |

S Fig 3B SH-SY5Y

| 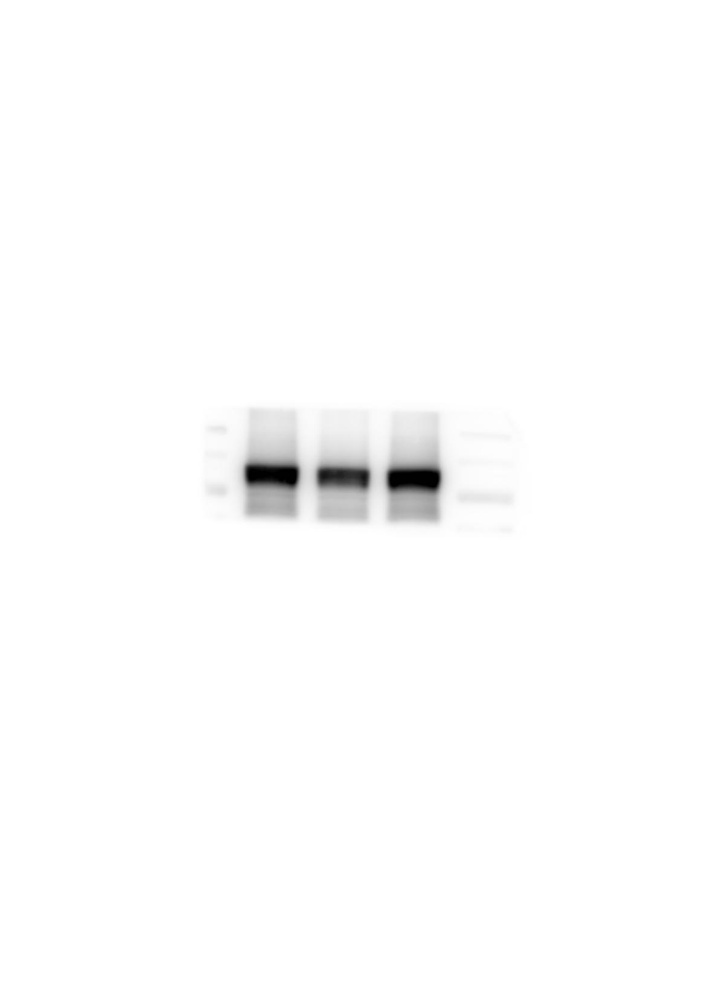 | FOXK1 97kDa |
| --- | --- |
| 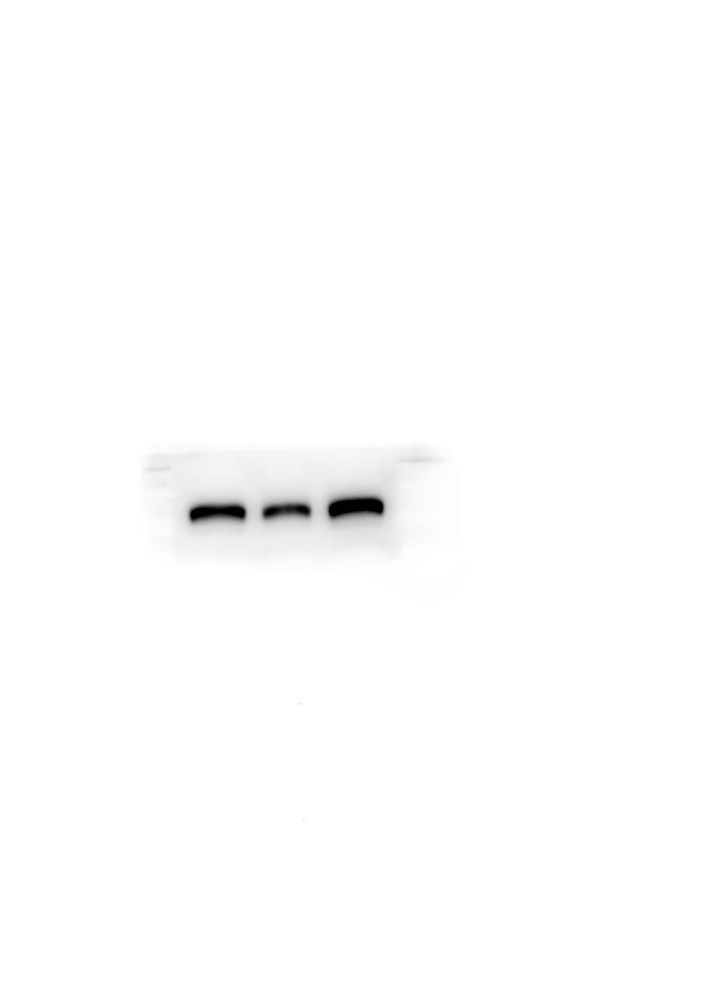 | HK2 102kDa |
| 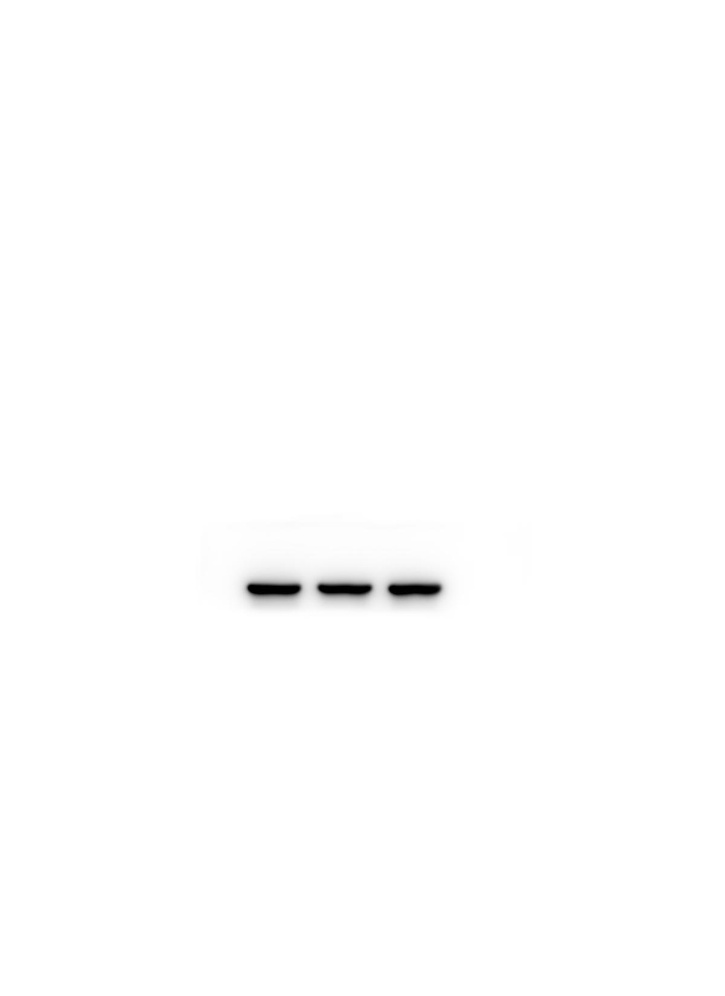 | β-actin 42kDa |
