# Supplementary material for: Adipose tissue-derived stromal cells enhance glycolytic metabolism in injured nerve cells via the FOXK1-HK2 axis for spinal cord injury repair
Source: J Transl Med. 2026 Mar 17;24:582. doi: 10.1186/s12967-026-07958-w (PMC13107693; doi:10.1186/s12967-026-07958-w)
Supplement: Supplementary file 3 — Supplementary Material 3 [file 12967_2026_7958_MOESM3_ESM.docx]

**Supplementary Table 1 Primer sequences for RT–qPCR**

| Primer name | Forward primer sequence | Reverse primer sequence |
| --- | --- | --- |
| β-actin | GCCGGGACCTGACAGACT | TGGCCATCTCTTGCTCGA |
| Hk2 | CTGGTTTCAAAGCGGTCGAA | AAGCAGGCGATCATATGCGA |
| Gpi | CCACCAAGGCACCAAGATGA | TGTGATGCAGACCATTCCGT |
| Slc2a1 | TTAATCGCTTTGGCAGGCGG | AAACCCATAAGCACGGCAGA |
| Eno2 | AACTTCCGGAATCCCAGTGT | AGGTGAGTCGAGGTGTTCTG |
| Tpi1 | CCAACCGCCTACATCGACTT | GCCAGGACTGATTTCCCCAG |
| Map2 | CATTTTGGTGCTGATCTCTCT | AGCTGGCCTTCTCCACAC |
| Gap43 | TCCTCTCCTGTCCTGCTCAC | TCGCCATAACAACACCAAGA |
| FOXK1 | CCCGTGTCCCGTTGTTTTTC | GCAACAGGTACGGACTTCCA |


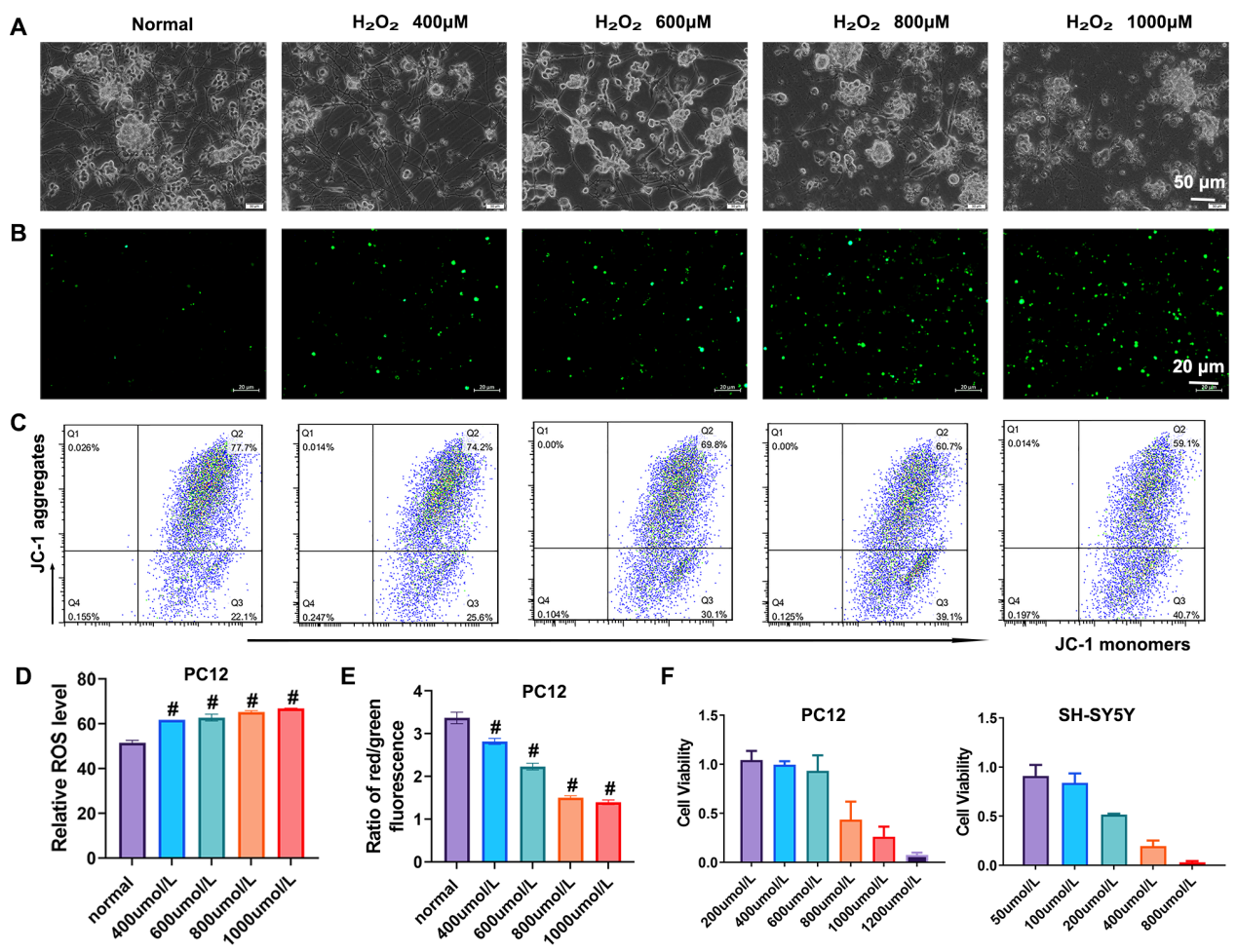


**Supplementary Fig. 1** Establishment of an oxidative damage model of nerve cells. **A** PC12 cells were treated with different concentrations of H₂O₂. **B, D** ROS levels were measured via DCFH-DA and analyzed for fluorescence intensity. **C, E** Flow cytometry analysis of the mitochondrial membrane potential in PC12 cells. **F** Viability of PC12 and SH-SY5Y cells treated with different concentrations of H_2_O_2_. # *P* < 0.0001, compared with the normal group; one-way ANOVA tests, Tukey’s multiple comparisons test.


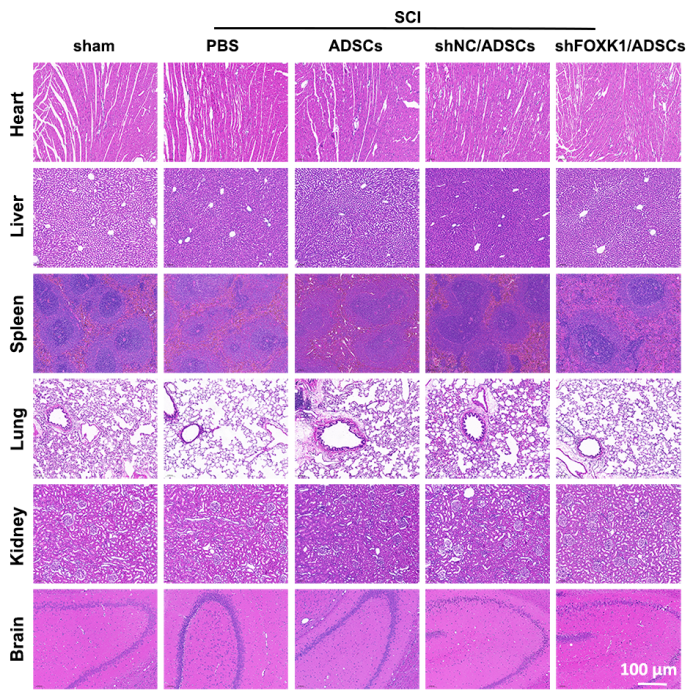


**Supplementary Fig. 2** ADSCs implantation has no toxicity in vivo. HE staining was performed on the heart, liver, spleen, lung, kidney, and brain of the rats in each group. Scale bar: 100 μm.


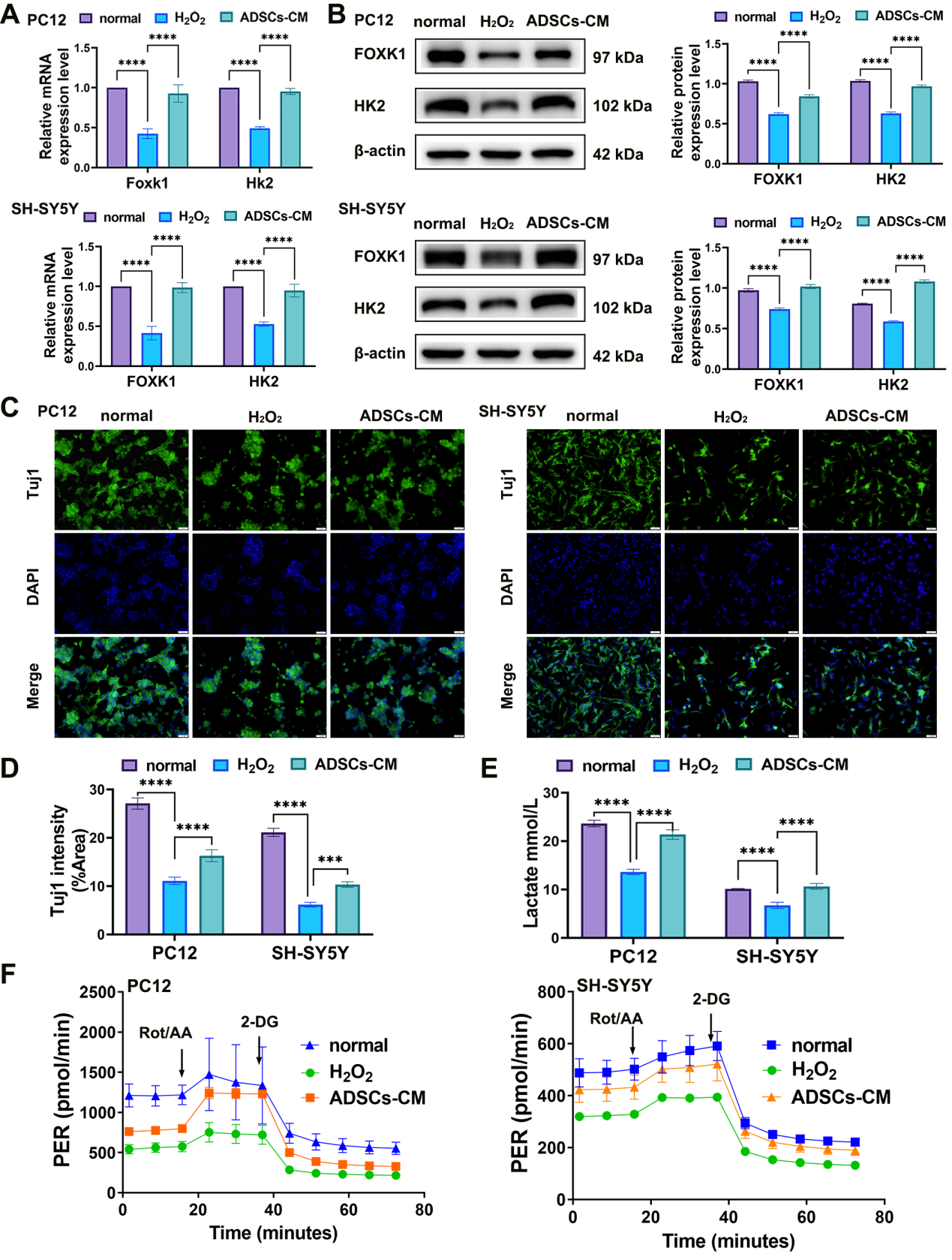


**Supplementary Fig. 3** ADSCs exert their effects via paracrine secretion. **A** RT–qPCR was performed to detect the expression of FOXK1 and HK2. **B** Protein expression was detected via Western blotting. **C–D** Immunofluorescence staining was performed to detect Tuj1 expression in PC12 and SH-SY5Y cells. **E** Detection of lactic acid in the supernatant. **F** A representative Seahorse glycolytic rate assay was used to assess cellular glycolytic metabolism.
